# A Survey on Wireless Wearable Body Area Networks: A Perspective of Technology and Economy

**DOI:** 10.3390/s22207722

**Published:** 2022-10-11

**Authors:** David Samuel Bhatti, Shahzad Saleem, Azhar Imran, Zafar Iqbal, Abdulkareem Alzahrani, HyunJung Kim, Ki-Il Kim

**Affiliations:** 1 Faculty of Information Technology, University of Central Punjab, Lahore 54590, Pakistan; 2 School of Electrical Engineering and Computer Science, National University of Sciences and Technology, Islamabad 44000, Pakistan; 3 Faculty of Computing & A.I., Air University, Islamabad 42000, Pakistan; 4 Computer Science & Engineering Department, Al Baha University, Al Baha 65799, Saudi Arabia; 5 Department of Computer Science and Engineering, Chungnam National University, Daejeon 34134, Korea

**Keywords:** wireless body area networks, wearable sensors, WBAN architecture, taxonomy, WBAN security, WBAN global market

## Abstract

The deployment of wearable or body-worn devices is increasing rapidly, and thus researchers’ interests mainly include technical and economical issues, such as networking, interoperability, security, power optimization, business growth and regulation. To address these issues properly, previous survey papers usually focused on describing the wireless body area network architecture and network protocols. This implies that deployment issues and awareness issues of wearable and BAN devices are not emphasized in previous work. To defeat this problem, in this study, we have focused on feasibility, limitations, and security concerns in wireless body area networks. In the aspect of the economy, we have focused on the compound annual growth rate of these devices in the global market, different regulations of wearable/wireless body area network devices in different regions and countries of the world and feasible research projects for wireless body area networks. In addition, this study focuses on the domain of devices that are equally important to physicians, sportsmen, trainers and coaches, computer scientists, engineers, and investors. The outcomes of this study relating to physicians, fitness trainers and coaches indicate that the use of these devices means they would be able to treat their clients in a more effective way. The study also converges the focus of businessmen on the Annual Growth Rate (CAGR) and provides manufacturers and vendors with information about different regulatory bodies that are monitoring and regulating WBAN devices. Therefore, by providing deployment issues in the aspects of technology and economy at the same time, we believe that this survey can serve as a preliminary material that will lead to more advancements and improvements in deployment in the area of wearable wireless body area networks. Finally, we present open issues and further research direction in the area of wireless body area networks.

## 1. Introduction

Wireless networks have become an indispensable part of social, defense and healthcare applications. These applications have received very swift support from different wireless network interfaces such as Personal Area Network (PAN), Body Area Networks (BAN), WiFi 802.11, WiMAX and cellular [1,2]. These technologies are extensively being used in healthcare systems [3]. In addition, they are also very benign in social gatherings, which can be envisioned from the popularity of wireless communication when friends or guests are sitting at a coffee cafe or hotel and share some information using WiFi or Bluetooth; passengers can pay for their tickets at a ticketing booth while connecting their mobile or smartphone to the electronic booth wirelessly. Similarly, it has also been observed that passengers traveling on a train or bus have demonstrated a need to have a hotspot (ad hoc network) of mobiles and smartphones for data sharing. In addition to all that usage, the application market of mobile and smartphones is becoming saturated day by day, and novel paradigms in mobile devices and sensors are arising in different dimensions of human life. These are basically the wireless wearable body area networks [4], which are assuring the improvement in the quality of human health, which was not possible for smartphones and mobiles to achieve alone. The terms “wearable”, “wearable devices”, “wireless BAN (Body Area Network)” or “wearable technology” are interchangeable. Similarly, wireless wearable body area networks and wearable wireless body area networks are also used interchangeably in the literature. These terms refer to small electronic or mobile devices, microchips, or other computing devices that possess the capability of communicating data wirelessly. Furthermore, these devices are fabricated into gadgets, clothes, or accessories that can be worn, patched on the body, or devices that can be implanted, injected or ingested in the body [5].

**Figure 1:**
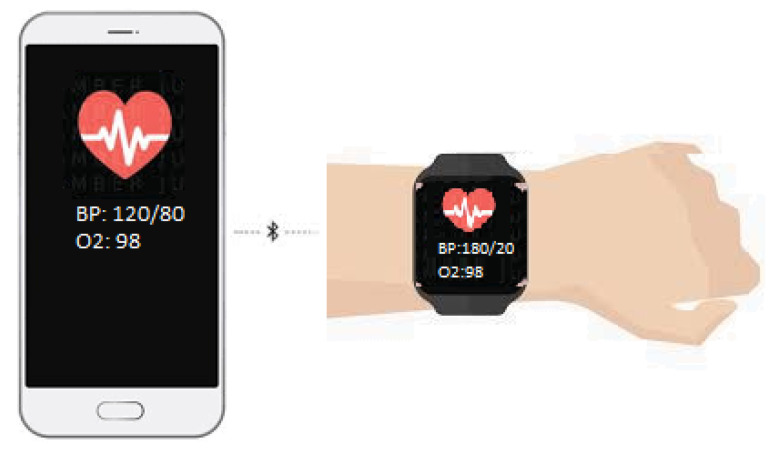
Health monitoring smartwatch [13].

The outstanding use of wearable/body-worn technology is giving rise to new dimensions in textile, sports, medicine, healthcare, and fitness applications, putting them into a bigger category of wireless networks known as Wireless Body Area Networks (WBANS) [6,7,8,9]. Smartwatches, wristbands, smart glasses, smart jewelry, electronic garments, skin patches, etc., are a few examples of such body-worn or wireless wearable devices [10,11]. They can easily be integrated with smartphones, laptops, or data servers using any of the wireless technologies shown in Figure 1.

Figure 1 shows how wearables monitor a person’s health and how the health data can be transferred to smartphones. There are a lot of videos available on YouTube that show how health monitoring smartwatches can be integrated with smartphones, mobiles, or laptops to monitor life-critical information. One such channel that can be found on this topic is “Jimmy is Promo”, which has uploaded a very good video tutorial for connecting a vital sign monitoring smartwatch to a mobile phone [12].

**Figure 2:**
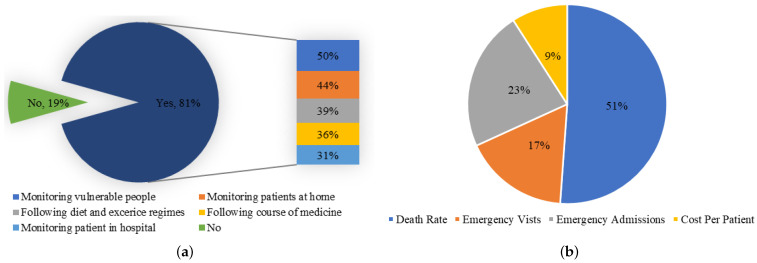
Wearable WBAN survey. (**a**) Usage and popularity of wearable/connected health devices [18]. (**b**) reduction in death, visits, admissions, and cost [16].

One of the best surveys on the consumer’s attitude toward the use of connected and wearable BAN health devices was carried out by Victoria Lambert [14]. This study was carried out on the people of the United Kingdom (UK) to assess how they see these devices being used in healthcare. It is a 2016 study, but it clearly predicts the future perspectives of the adoption of wearable BAN devices at individual, group (troops, BBN, etc.), and organization (hospital) levels. It shows that more than 80% of people want to have wearable BAN devices in healthcare systems. This study shows that different people are using these devices for different purposes, as shown in Figure 2a. Presently, it can be seen from the data how extensively these devices are being shipped and used.

The use of wearable BAN devices has been increasing very rapidly for the last couple of years. Different reports have shown the present and future trends of usage of these devices. For instance, a 17% CAGR (Compound Annual Growth Rate) of the wearable BAN market is expected between 2021 and 2025, which was recently reported by Business Wire (UK) [15]. A separate section (Section 3) is dedicated to a global market of wearable BAN devices whose increased popularity and adoption have resulted in a reduction in mortality rate, patient visits and admissions to emergency departments, and costs per patient. Figure 2b shows quite encouraging figures of these parameters in terms of reduction in the percentages of the above-mentioned variables [16]. It affirms the well-being of human health due to the use of wearable BAN devices at personal, mobile (ambulance, travel, troops), and organizational (hospital) levels [17].

### 1.1. Research/Survey Methodology

Since this manuscript is a survey article, it may require a lot of citations and references from other journals, conference articles, books, lectures, audio/video tutorials, business reports, blogs, websites, and published and even unpublished material. We believe in a fair and unbiased research methodology. We tried our best to keep ourselves unbiased in citing and referencing the material and include justifiable content regardless of the fame and ranking of the publishers. Our study does not promote any publisher or journals as sometimes observed and heard in research groups. However, the major keywords used to search the contents were “wearable devices”, “body-worn devices”, “wireless wearable body area networks”, “wireless technologies used in WBAN”, “wearable wireless technologies”, “wearable wireless protocols”, “WBAN security threats”, “WBAN wireless security protocols”, “state of the art wearable security protocols”, “WBAN and Wearable regulatory bodies”, “wearable Compound Annual Growth Rate”, “wearable CAGR”, “wearable WBAN challenges”.

### 1.2. What Questions Does This Survey Answer?

This survey efficiently answers the following questions.

1. What is a wireless wearable body area network and its communication architecture?
2. What sensors are used in Wearable Wireless BANs, and what end products and solutions are available in the market?
3. What are the potential wireless technologies used to develop WWBANs, and what security threats and control measures are available?
4. What does the future hold, and what major challenges do the wireless wearable body area devices have to face?
5. What are the major projects and research directions in this area?

### 1.3. Contributions

We believe in contributive and productive studies, which is why we have put in the best of our efforts to make this article productive and cultivatable for the broad spectrum of viewers. We have gone through many survey articles; the majority of them focus on a limited set of WBAN perspectives. For example, Hajar et al. [18] discussed WBAN security, privacy, and energy harvesting concerns but did not discuss WBAN sensors, medical and non-medical devices, growth rate and regulatory bodies. Some articles, such as that of Kim et al. [19,20], were found to only emphasize the dynamic resource management perspectives of WBANs and the resource allocation at the MAC layer. Asisha et al. [21] discussed the WBANs MAC layer. Propagation characteristics and channel modeling for Internet of Bodies (IoBs) using human body communication are reviewed by Kadir et al. [22]. Therefore, the major contributions of this study are listed below, which help a prospective reader to judge the productivity and usefulness of this study. Zhong et al. [23] only bring WBANs technical requirements and challenges into discussion.

1. During our study on WBANs, we found that a majority of the reports focus on only a particular set of viewers as discussed above.
2. The present article addresses maximum perspectives of wireless wearable body area networks such as architecture, components, sensors, devices, health systems, wireless technologies, security concerns, projects, regulations, and most important, and Compound Annual Growth Rate (CAGR).
3. A special section is added in this survey that focuses on the emerging WBAN technologies, such as energy harvesting, human body communication, quantum computing, graph neural networks (GNN), artificial intelligence (machine and deep learning), blockchain, and digital twins. Such a section is rarely seen in surveys or review articles.
4. Since the article is interdisciplinary, technical terms of a particular field are briefly explained on the spot so that a specialist of one field may understand the concepts of another without repetitive switching from this article to another.

## 2. WBAN Technical Issues

### 2.1. WBAN Architecture

WBANs consist of a control unit and sensors. These sensors might be implanted in the body (implanting medical devices IMB) or they might be wearable, body-worn, or body-patched. These sensors can communicate with each other or with the help of a control unit using a wireless link. They can monitor vital health signs such as “Body Temperature”, “Blood Pressure (BP)”, “Respiration Rate (RR)”, “Skin Perspiration”, “Blood Glucose”, “Heart Rate (HR)”, “Oxygen Saturation”, “Electrocardiogram (ECG)” etc. The sensors can also capture similar parameters from the environment, such as pollution, which is directly linked to cardiac and pulmonary diseases [24].

**Figure 3:**
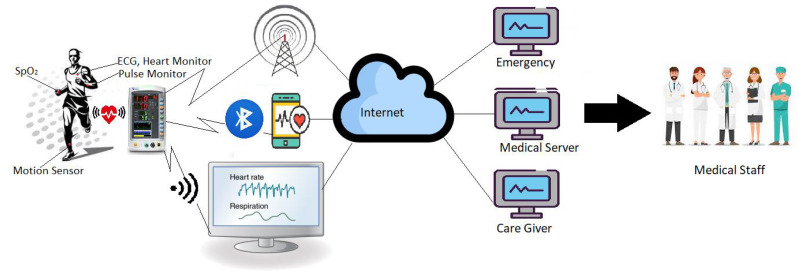
Wearable body-worn motivational model [11].

In the sequel to the figure, Figure 3 shows how data from a smartphone/gateway/ coordinator can be sent to a centralized repository (medical server) for medical and paramedical staff to take necessary decisions. This server might be cloud-based to provide 24 h service to different stakeholders [11]. A typical architecture of a wireless wearable BAN is shown in Figure 3. In this architecture, the communication system is divided into three layers as follows [25].

*Layer-1:* An intra-BAN devices communication tier, where different sensors collect body vital signs such as BP, HR, sugar level, ECG, etc., and they pass it to the control unit or even directly to the gateway depending upon the wireless link.

*Layer-2:* This layer is a setup of inter-BAN communication in which the body coordinator sends information to a personal server that might be a smartphone, mobile, PDA, or laptop.

*Layer-3:* From the personal server, the health information is accessed by different medical or paramedical entities such as caregivers, doctors, or health systems/subsystems using an internet connection (Satellite, GPRS).

The evolutionary perspectives of the discussed architecture can be reviewed from the article published by Aleksandr et al. [5], which provides a very comprehensive overview of state-of-the-art wearables, their communication, history, and challenges.

#### Components

The following is a list of components that are necessary to make a wireless wearable body area network [25,26].

*Sensors* are devices that take some input from physical surroundings and respond to it [27]. Biosensors are concerned with the vital sign monitoring of living beings [26]. They may be used to collect health information such as BP, HR, ECG, and body temperature and transmit it to a BAN control unit [28]. The requirement of body sensors is that they must adhere to lightweight, low power consumption and fast switching between sleep and wake-up mode for energy saving. Moreover, the material used to make sensors should not be harmful to the human body. *Controller Unit or Sink* receives data from the sensor and transmits it to the gateway or personal server. It can also generate alerts upon receiving abnormal values of vital signs [28]. *Antena* is responsible for the basic transmission and reception of data. It must be small, lightweight, human-body favored, and operate at high frequencies. The design of a flexible, small size and power-efficient antenna for wireless wearable body area networks is proposed by Karthikeyan et al. [29]. Similarly, as a key component, a high-performance UWB antenna for WBANS was also designed by Yazdandoost et al. [30]. The authors used two layers of a substrate to nullify the effect of a human body on the performance of a proposed antenna. Other intelligent antennae designs for wireless wearable body area networks are proposed by Zhang et al. [31,32]. *Communication* is the major component of WBANs. Wireless mode of communication is used by BAN nodes to make transmissions, but it must lie in those frequency bands that are not resilient to interference caused by the co-existence of similar networks [26]. Therefore, there should be a barrier that can avoid mixing frequencies while operating in real-time setups. The communication protocol must ensure seamless *handover* when the BAN user leaves the covering area of one gateway and enters another. Some of the techniques proposed for this component are [33,34,35]. Moreover, a detailed survey on vertical handover techniques for integrating wireless body area networks with other heterogeneous is documented by Saurav et al. [36]. *Security* is another key part of WBAN communication that must ensure the data do not leak and no malicious entity hacks or cracks it [26]. Strong access control and encryption techniques need to be applied to BAN data when they are transferred from the body to some centralized repository. *Personal Server or Gateway* is another important component that resides between BAN controller and the cloud, i.e., internet. It receives data from the BAN control unit and passes it to the cloud or centralized medical server [28]. *Internet link* is also a crucial component that is responsible for securely (use of VPN, IPSec, SSL, TLS, etc.) transferring health information from the local gateway to a remote centralized server. The link should be reliable in terms of security, availability, and turnaround time [37]). There must be a component called *alerts mechanism* that should generate alerts in different critical conditions, such as reception of abnormal vital sign signals or when the battery power critically goes low, etc. [28]. There has to be a *medical server* (centralized repository) where data could be stored and retrieved at any time by caregivers, physicians, or other data consumers [28]. With only *BAN users* (such as patient, sportsman) and *medical staff* (such as caregivers, physicians), the BAN is incomplete. Therefore, all the components discussed above are used to connect BAN users and medical staff.

### 2.2. Types of Sensors

**Figure 4:**
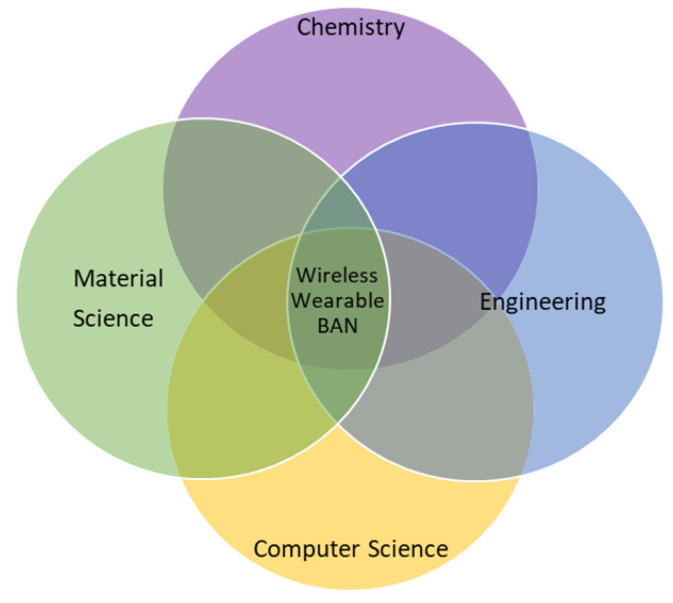
Wireless wearable BAN field.

Wireless Wearable BAN Sensors are the product of collaborative and mutual research efforts of researchers belonging to different fields of science and engineering; Figure 4 represents this well. Each sensor in a wireless wearable body area network is completely provided with communication capabilities and can act as an autonomous device [25]. Wireless wearable BAN architecture that is used to send health information to remote medical servers can have different types of sensors. In this study, they are discussed on the basis of implantation and their functions. The discussion about them is equally beneficial for the wireless wearable BAN user, practitioners, engineers, and designers.

On the basis of implantation, wireless wearable BAN sensors are categorized into five main types to assure the success of a healthcare system [25,38].

#### 2.2.1. Type-I Implantable

These are sensor nodes that are implanted inside the human body, such as pacemakers (e.g., Medtronic Micra) [39,40]. The importance of these sensors can be understood from research efforts being made to help blind people to see [41]. One of the fruitful outcomes of this effort is the retina prosthesis chip, which is implanted inside the human eye, and persons who are visually impaired may be able to see at a reasonable level [42]. *Pacemakers* are the most widely implanted devices in the human body under the chest or abdomen. The sensors of pacemakers are used to monitor cardiovascular irregularities. Medtronic Micra is one of the examples of pacemakers that have quite a long life (12 years) and can be scanned by MRI (Magnetic Resonance Imaging). *Intra-Cranial Pressure Monitoring Sensors* monitor the pressure that rises up due to the irregularities of cerebrospinal fluid, head injuries, or as a consequence of cerebral edema. These may lead to severe brain damage, nervous breakdown, or death. The most popular sensor used implanted in the human body for detecting the irregular changes in the value of ICP and IOP are MEMS pressure sensors and RF oscillators. Both types operate at 2.4 GHz. IOP stands for Intra-Ocular Pressure whose irregularity may cause intraocular pressure, which is carefully regulated, and disturbances often result in issues such as glaucoma, uveitis, and retinal detachment [43]. Glaucoma is a set of eye conditions that may break an optic nerve; it is often caused by high blood pressure and may lead to vision loss. Uveitis is an eye inflammation that causes glaucoma. Retinal detachment is an emergency eye condition in which the retina becomes detached from blood vessels, stopping oxygen supply to it. *Cardiovascular Pressure Sensors* observe the narrowing of the blood artery even after angioplasty, which is called restenosis. Continuous monitoring of chronic blood pressure that may cause heart failure, hypertension, or restenosis is very necessary. One of the recent developments in this regard is the FDA-approved chronic blood pressure monitoring device from CardioMEMs that helps detect heart failure. In addition to this, other recommended devices for chronic blood pressure monitoring are MEMS capacitive sensors, surface acoustic wave resonators, and accelerometers. *Neurosensors* have received a lot of interest from the research community for dealing with addiction, epilepsy (brain disorder), Parkinson’s (neurological movement disorder), and Alzheimer’s (gradual loss of memory) disease, etc. One of the reported cases is the RFID-inspired neurosensor considered for a wireless brain–machine interface, which is battery-less and the energy storage is accomplished using the concept of RF energy harvesting [44]. Similar research work is presented by Xiang et al. in which authors have proposed and tested a self-powered brain machine that harvests energy from the movements of a human body part and sends a signal to the brain to cease some action [45]. *Glucose sensors/meters* are mostly referred to as amperometric sensors. Glucose sensors are meant to determine the blood sugar level of a person while playing an important role in dealing with diabetes mellitus. Most people in their homes use a finger-pricking method to test their blood sugar level. However, with the progression of research in wireless communication, computer science, engineering, chemistry and material sciences, the dream of under-skin wearable devices has become a reality [46]. These sensors can be used for 90 days [47]. The registered devices being used for continuous monitoring of sugar levels in the blood are GlucoWatch^®^ Biographer, CGMS^®^ and GlucoDay^®^ [48]. There was a breakthrough in wearable smart textiles with the production of lightweight and mechanically robust wearable electronics that are based upon bio-inspired muscle fibers [49].

#### 2.2.2. Type-II Ingestibles

These sensors are small in size and can be ingested similarly to pills. These sensors are mostly used in ingestible capsules that are disposable and used to diagnose the correlation between ingested pharmaceutical drugs and physical activities of body responses such as heartbeat, quality of sleep and blood pressure, etc. [39]. *Imaging capsules* are the sensors that are passed through the gastrointestinal tract as disposable capsules. These can provide real-time data in the form of images or videos about the small bowel, gastrointestinal tract, esophagus, and large intestine at a rate of a maximum of 2 Mbps. Transmitted data by these imaging capsules can be used for different diagnostic purposes. For example, these devices are one of the best alternatives to aggressive endoscopic procedures, which may cause the generation of different gases in the human body (gut) revealing a strong correlation to gastrointestinal disorders [39].

#### 2.2.3. Type-III Injectables

Nano-technology is now going to bring a great revolution in bio-medical technology. This is a predicted future of biomedical devices with such a small size that would allow them to be injected with the help of a syringe [39]. *Injectable micro-sensors* are very small-sized sensors with a thickness of a few human hairs and the length of a rice grain. Recently, a demonstration of injectable bio-sensors was given by Profusa Inc. [50]. In their demonstration, they injected bio-sensors called Lumee Oxygen sensors using a needle. The injected sensor has the capability of observing oxygen levels in the tissues around it [39]. *pH sensors* have significant importance in monitoring tumor growth and the effectiveness of anticancer treatments. American scientists have developed pH sensors that are based on nanocrystal quantum dots and operate in the human pH range. These are injectable devices that can monitor the growth of tumors on a real-time basis [51].

#### 2.2.4. Type-IV Patchable: Body Surfaced/Patched

Sensor nodes that can be patched on the surface of a human body in their direct contact with it for establishing the communication network of on-body devices are termed as patched or surfaced wearable or simply patchable. The attraction of patchable devices is increasing in the biomedical industry due to their interfacing with skin on a cellular to molecular level. They are spatiotemporally precise in terms of bio-sensing for diagnostic and therapeutic purposes [52,53]. High blood sugar is a disease, but if monitored regularly, the chronic effects can be mitigated. Patchables are playing a very key role in achieving this [54]. A very promising study has been carried out on measuring the effects of ultra-violet radiations on the body using patchables [55]. A very brief but smart study on patchable sensors can be seen in [56,57]. This setup is used for providing an ambulance-based health monitoring system for a person [40].

#### 2.2.5. Type-V Proximity-Based Devices

Sensor nodes that are installed in close proximity (5–10 cm) to the body surface, e.g., smartphones and smartwatches, help in monitoring body fitness, where the values and results of vital signs are displayed on smart devices. Sweat-powered wearable electrochromic biosensors are one great contribution that provides a non-invasive way of monitoring lactate concentration (high means lack of oxygen) in the body [58]. WBAN devices are very diverse in nature. To monitor the same life sign in humans, they can be found in different forms such as implantable (in-body), body-surfaced (on-body), and wearable. This provides a great choice for a person trying to select a health monitoring sensor for a particular vital sign observation [59].

**Figure 5:**
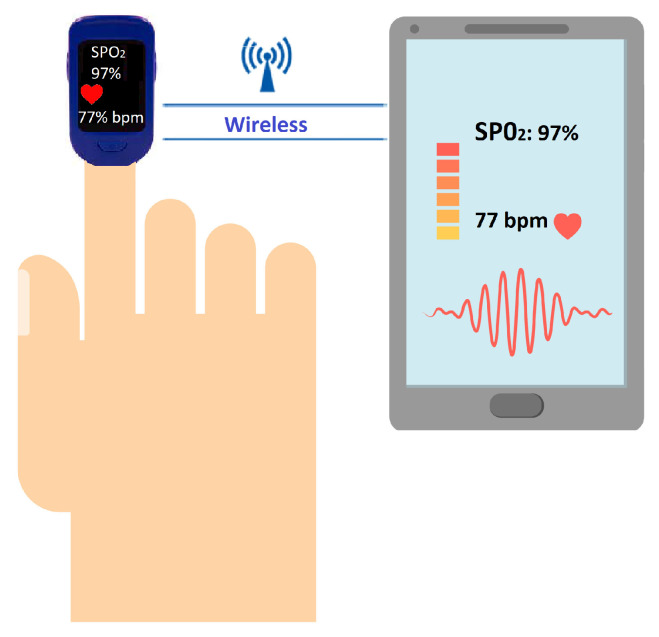
Wellue^®^ O2^TM^ Ring—continuous oxygen monitor [63].

*Oximeter* sensors are used to find the oxygen absorption (saturation) level in body cells. Devices make use of the human fingertip to find the values. Oxygen absorption level is one of the critical vital signs that determines how well the oxygen is being provided to body cells by the red blood cells to keep them alive [60]. Low oxygen supply turns the red color of the tissue to blue, which means their life is shortening. It also greatly damages the functionality of your brain. The World Health Organization (WHO) has also provided a very good tutorial on using an oximeter on its website [61]. However, the detailed principles of the oximeter are beyond the scope of this study but can be retrieved from [62]. A well-known example of this device is the Welue O_2_ Ring Oxygen Monitor, which is shown in Figure 5.

#### 2.2.6. Type-VI Textile Structured Devices

These are the structures and materials that sense and react to environmental factors. They are being used in the textile industry, telecommunications, biochemistry, bio and information technology, etc. Textile-based sensors have obtained significant attention in smart wearable technology. They are low-cost, comfortable, and flexible. They can be used remotely to observe the physiological parameters of the human body [64]. The development of textile-based energy storage devices are outstanding research works that can be used in wearable technology to address the power consumption issues [65]. Advances in wearable electronics are going to replace spacey, non-flexible and uncomfortable wearable electronics [66]. In addition, printed textile batteries are also being researched for wearables, which will mitigate energy problems of these devices [67].

This diversity is summarized in Table 1. In addition to this, one of the surveys reports that based on transducing mechanisms of biosensors, we can categorize them into optical and thermal detectors, resonant, Ion-Sensitive FETs (ISFETs) and electro-chemical biosensors [68]. Another survey that sheds light on the biosensor and their state-of-the-art development is carried out by Naresh et al. [69]. These articles are good enough to review.

### 2.3. Wireless Technologies

Wireless body area networks are found in the form of sensing devices implanted on or inside the body of a living organism. These devices are extensively used for monitoring vital signs of the body using wireless transmission protocols. However, the researchers agree that Wearable WBANs are different from the WSNs, and their requirements vary from the WSNs as well. This is the reason that present protocols that are designed for WSNs are not compatible with the needs and requirements of wearable WBANs. These differences are: the number of nodes deployed in WSNs is considerably higher than the WBANs, and there are no duplicated or redundant nodes placed in WBANs but deployed in WSNs. Such differences create communication complications at different layers of the network protocol stack. Similarly, there are hard and fast limits on device size and power consumption in the case of WBANs as compared to WSNs. In addition to that, because WBANs are concerned with the collection and transmission of critical life information, the reliability, security, throughput, and delay of such scenarios are of high importance compared to other wireless network applications [70]. One of the distinguished aspects of wearable WBAN devices is that each device is deployed or implanted to perform a different task. There is a high level of heterogeneity in WBAN, which ultimately asks for different designs and implementations, whereas in WSNs, the nodes are supposed to perform tasks of a similar type or nature, showing a great homogeneous feature. Application of body-worn devices may include capturing of health data and transferring it to a processing server, collecting acceleration information to control robots, or giving commands and instructions to the game controller or console.

Looking at the above discussion on the nature of the WBAN devices, it can be concluded that the selection of wireless technology to be inducted for wearable or WBAN devices is very critical. Bandwidth, power, range limitation, and the nature of the application of wearable WBAN devices determine the type of wireless technology to be decided. Therefore, no single wireless technology satisfies all wearable WBAN applications and scenarios [71]. The most dominating wireless protocols in the WBANs, wearable or body-worn industry are Near Field Communication (NFC), Bluetooth Low Energy (BLE), Bluetooth Classic, Zigbee, 802.15.6 WBAN, WiFi, LoRa, ANT, ANT+, and Cellular [70,71,72,73].

**Figure 6:**
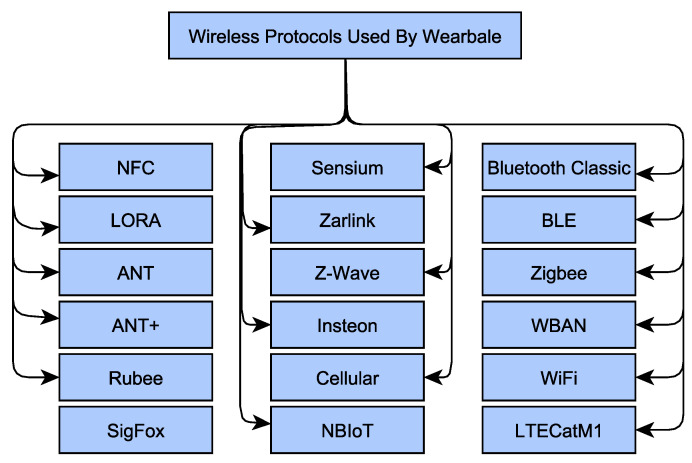
Wearable, body-worn wireless protocols.

Figure 6 gives a quick glimpse of all these protocols. We would like to mention that these wireless technologies can be considered the candidates for intra-BAN (e.g., UWB, Bluetooth, Zigbee, WiFi), inter-BAN (Bluetooth, Zigbee, WiFi, Universal Mobile Telecommunications System (UMTS/GPRS), and/or beyond-BAN (GPRS/UMTS, Internet, WiFi, Cellular Networks, multi-hop 802.11 internets, Global System for Mobile Communications (GSM)) communication [74].

#### 2.3.1. NFC

NFC stands for Near Field Communication and highly suits the wearables that are extremely power-constrained and required to transfer data for a range of a few centimeters, that is, about 4cm-10cm. The NFC device operates in two modes, i.e., passive and active. In passive mode, the NFC does not consume any energy, whereas in active mode, it draws energy almost equal to BLE. NFC-enabled smart devices can be paired with body wearers such as smart suits and jackets to process cardless payments. Similarly, they can be used to open doors and even transmit health information. NFC is also being used extensively in smart jewelry, wrist-worn wearables, E-Skin and E-tattoos [72]. Attacks on NFC-shared secret keys and certificate-based solutions have been proposed in [73].

#### 2.3.2. ANT

ANT is one of the emerging wireless protocols for WBAN health monitoring applications. ANT stands for “Advanced and Adaptive Network Technology”. It is suitable only for low-speed and low-power WBAN applications. Several health monitoring sensor manufacturers are using this protocol. Different wearables are also operating in ANT, Bluetooth 4.0 or WiFi mode [72].

#### 2.3.3. RuBee IEEE 1902.1

RuBee is one of the strong contestants of WBAN wireless protocols due to its low energy consumption, long-life battery, security, and stability. It sends small-size data packets of 128 bytes using long-wave magnetic signals. It makes use of low-frequency waves, i.e., 131 kHz, and is considered to be a slower technology compared to other wireless communication technologies, such as 802.11. The frequency at which this wireless technology works is not vulnerable to attenuation induced by metallic or liquid substances. This is the reason why RuBee devices can be sustained in harsh environments [74].

#### 2.3.4. Sensium

**Figure 7:**
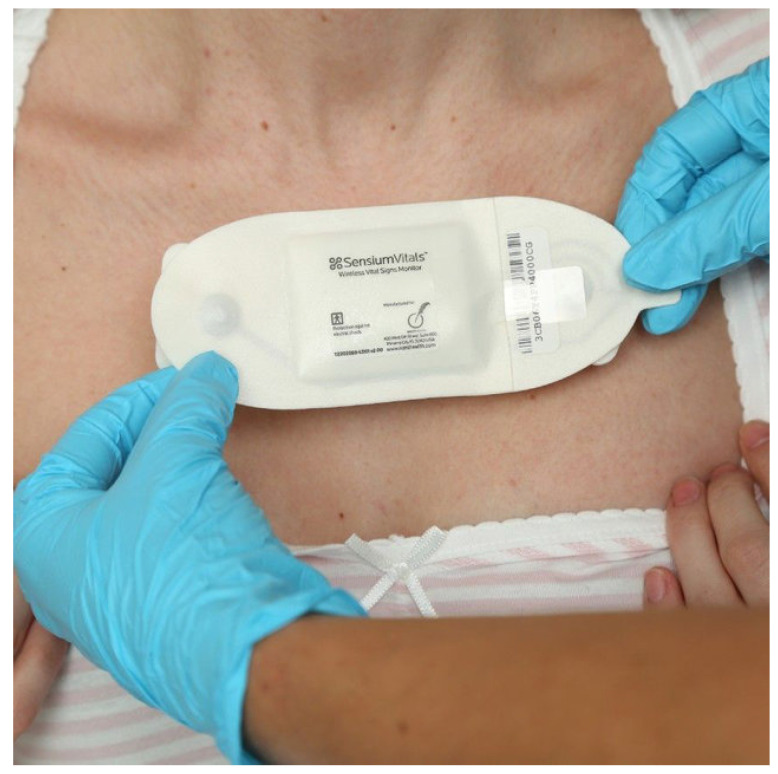
Sensium patch is being placed on the patient’s body [57].

Sensium is a low-power on-body technology for steady monitoring and observing of patient health [75]. It can send information to smartphones, mobile phones, laptops, and PC devices wirelessly, which is sent to a medical entity (medical servers, doctors, paramedical staff) for further action. Sensium sensors become alive only when they have reached a time slot to transmit data; otherwise, they keep themselves in sleep mode to save energy [74]. Presently, Sensium is one of the most widely adopted patient health monitoring systems. Figure 7 is the real Sensium copyright picture in which the Sensium patch is being placed on the patient’s body for vital sign monitoring. We reproduced this picture from the open access article [57]. From this figure, the importance of wearable WBAN devices in terms of patient health monitoring can be imagined. This acted as one of the most promising technologies for remotely treating patients with COVID-19, where people were required to stay at home as much as possible.

#### 2.3.5. Zarlink

Zarlink is energy efficient, one of the pioneering wireless protocols used in health care and diagnostic applications, and is equally good in implanting cases of WBANs. The cameras swallowed by a patient for the purpose of diagnosis and deep investigation of the gastrointestinal tract usually use Zarlink technology. The Zarlink-based camera is also used to send back videos of the tract in a timely manner with good quality [74]. It can operate in one of the two modes, i.e., IMD (Implantable Medical Device) mode and base mode. It consumes minute power (μW) in IMD mode and was announced as an energy-efficient device [76].

#### 2.3.6. Z-Wave and Insteon

Both are open and proprietary wireless mesh technologies developed for home automation. Z-wave operates at 2.4 GHz, whereas Insteon leveraged the features of powerlines and the 900 Mhz ISM band [77]. It is used for smart homes but can be discussed as a candidate of WBAN wireless protocol for being the competitor of X10, UPB, Z-Wave, ZigBee, Bluetooth, BLE, and WiFi. Z-Wave is the next-generation wireless technology that makes use of low-powered and reliable waves, which have the capability of passing through walls and solid objects. It is believed that these are quite good protocols for wireless body area networks that are based on these technologies [74].

#### 2.3.7. Bluetooth Classic

IEEE 802.15.1 Bluetooth is one of the successful wireless technologies for short-range communication with high security [78]. The compelling feature of Bluetooth is that it permits a large variety of Bluetooth-supported devices to make connections and establish communication with one another. One active device called a master can connect itself to seven active slave devices in a single ad hoc network called a piconet. It connects to 255 inactive slave devices, also called parked nodes, which can be activated by the master at any time. Parked nodes are kept in low power mode for energy conservation. Bluetooth-enabled devices operate in the 2.4 GHz band [79]. Their transmission range may vary from 1 to 100 m, and the maximum data rate could be 3 Mbps. Bluetooth is observed to be used in different areas of telemedicine and e-health systems. In addition, high bandwidth requirement, high energy consumption, longer pairing times, and a small number of devices in the network make this technology less suitable for wearable WBANs [70].

#### 2.3.8. Bluetooth Low Energy (BLE)

BLE is an advanced and improved version of the classic Bluetooth that claims to be quite suitable for the WBAN devices due to its fewer energy requirements and low duty cycling rate [71]. It was designed to connect tiny devices with mobile devices, but due to low energy consumption, it is considered to be a great wireless technology for wearable and healthcare devices. Its synchronization time is less than the classic Bluetooth, and it uses adaptive frequency hopping and fewer channels for pairing. It also operates in the 2.4GHz (ISM) band as that of classic Bluetooth, which may cause interference with other devices operating in the same band. It suits the scenarios where a high data rate is required for a short period with low power consumption such as peer–peer communication between the wearable sensors and the access point (AP) or between WBAN devices connected in an ad hoc mode or between two WBAN devices or between a WBAN device and the mobile, laptop or personal computer. However, it cannot be ignored that in critical scenarios where frequent and steady reporting of health parameters is required, BLE seems to lack behind in the race of other favorable wireless technologies for WBANs devices [70]. In addition to this, BLE has not become common among the different vendors of wireless technology.

#### 2.3.9. Zigbee

Zigbee is the combination of two technologies, Zigbee Alliance and the IEEE 802.15.4. Zigbee Alliance defines the application layer, i.e., network, security and applications software. The IEEE 802.15.4 module specifies the MAC and physical layer. Every Zigbee network consists of three types of devices, which are coordinator, router, and end-device. The job of a coordinator is to start and manage all sorts of connectivity and routing operations [78]. Its transmission range is a few hundred meters, but power consumption is higher than BLE [71]. This technology is highly suitable for applications where the device needs to run for several years [79]. The energy consumption of ANT > Zigbee > BLE [80]. However, at the same time, BLE has a very low data rate and operates in 868 MHz, 915 MHz, and 2.4 GHz bands, which may cause interference with devices that are already operating at 2.4 GHz. It is not suitable for large-scale real-time wearable WBAN scenarios, but it is good for single-user personal use. The Zigbee rate of suffering from channel fading is high, implies long delays in transmission of health data, which declares it unsuitable for critical WBAN application and is required to carry urgent data without such delays [70].

#### 2.3.10. Ultra-Wideband (UWB)

UWB has been standardized by IEEE 802.15.3, providing wider bandwidth for high data rate WPAN applications designed for transmitting multi-media data (images and videos) [81]. The high data rate features of UWB make it very supportive for a number of wearables and body sensors that are used for remotely monitoring the patient’s health. These are not the only features that make it a good candidate for wearable and BAN applications, it is also a power-efficient technology. Low energy consumption increases the life period of wearable and BAN devices while helping to transmit more data. UWB can be applied in two forms, multi-band OFDM (Orthogonal Frequency Division Multiplexing) UWB and IR (Impulse Radio) UWB [82,83]. IR-UWB is more suitable for low-resource wearables and WBAN devices because it consumes less energy compared to multi-band OFDM UWB. IR-UWB uses a very short pulse to transmit data, which makes it energy efficient and very suitable for short-range WBAN data transmission [25].

#### 2.3.11. LORA

LORA stands for Long Range, which is long-range low-power wireless communication technology. It was developed by Semtech and has become a de-facto standard for IOT networks [71]. It is extensively being used for low power wide area networks (LPWAN). The open source feature of LORA enables organizations to build an autonomous, low-power, and long-range communication system at a very economical cost [84]. The standard LORA follows security features, as shown in Table 2.

#### 2.3.12. WBAN IEEE 802.15.6

The first standard that was designed especially for the WBANs was IEEE 802.15.6. In some cases, WiFi technology is being used for information acquisition in such a way that fixes the direct communication between the sensors (for data acquisition) and directly transfers it to mobile or PC without the intervention of any intermediate relaying or routing devices [78]. Most medical, non-medical wearable and on-body WBAN devices have adopted this standard for communication. Similarly, different wearable wireless devices have been attracted toward this technology due to its support for a wide range of data rates, low transmission power, low range, and connecting a quite reasonable number of wireless devices (256) on the body in a single WBAN. It operates in different frequency bands, for instance, Narrow Band, Ultra-Wide Band, and 2.3 GHz and 2.4 GHz bands. No doubt, this standard fulfills most of the requirements laid out by the WBANs. However, the standard is unable to fit in the emerging scenarios of WBANs such as running, sleeping, jogging, sitting, laying, etc. In addition to this, BANs substandard HBC (Human Body Communication) is not a promising wireless communication technology for the WBAN applications, which are required to transmit audio or video data [70,79]. Similarly, some of the bands, such as UWB, can be used only by authorized users. Now, the research community has united on this point that the 2.4 GHz band is the most appealing one for wearable, body-worn, and WBAN medical applications. In addition, all other properties are discussed in Section 2.3.13, and it has the capability of guarding against channel interference. The IEEE 802.15.6 working group has defined Physical and MAC layer modules of the WBANs that are ultra-low power, low cost, less complex, and short range. They provide communication services inside and over the human body. IEEE 802.15.6 central node can interact with Bluetooth, BLE, ZigBee, UWB, NFC, RFID, Zarlink, RuBee, Sensium, Insteon, Z-wave, and ANT/ANT+ [86].

#### 2.3.13. WiFi

IEEE 802.11 named WLAN or WiFi is a widely adopted wireless technology in mobiles, smartphones, tablets, PCs, laptops, PDAs, etc. [71,79]. We believe 2.4 GHz is also the most suitable wireless technology to be adopted at a large scale for wireless wearable and WBAN medical applications [70]. It is highly appropriate for large data transfers at higher bit rates [78]. However, at the same time, we cannot ignore the energy consumption of this technology. Fortunately, at the same time, a modified version of WiFi called Low Power WiFi has been researched in wireless sensor networks. This low-power WiFi has been outfitted with reduced transmission power, low-duty cycling operation, and optimized energy consumption features. WiFi is already positively acknowledged for its security and QoS traits. WiFi technology is commonly observed in homes, marts, hospitals, banks, and handheld smart devices. Therefore, it can be concluded that the integration of these low-power WiFi-enabled devices with the networks in their vicinity is quite compatible and easy. WiFi is the only wireless technology, which in addition to lower transmission power and energy optimization capabilities, supports multimedia applications such as live audio/video streaming, which is not possible with NFC, Bluetooth, Zigbee, LORA, and ANT. The prime logic for inducing low-power WiFi in wireless sensor networks is to bring about the concept of the Internet of Things. The materialization of this concept affirms WiFi to be an encouraging wireless technology for WBAN or body-worn devices [70].

#### 2.3.14. Cellular

As far as cellular networks are concerned, smart wearable WBAN devices can directly connect and communicate with the cellular network instead of engaging themselves in bridging to communicate with them. At the same time, it can not be ignored that previous network technologies were more human-oriented. However, they have to face a lot of issues and challenges in the cases of wearable WBAN devices, especially when applications vary from low-end vital sign monitoring to high-end virtual or augmented reality applications. An example of such an application is virtual/augmented reality helmets for entertainment [87]. These applications require data rates in Gbps, which the present wireless technologies are not supporting, except the 802.11ac, which claims a data rate of 1GBps. The 5G wireless technologies have promised a data rate of Gb per second. However, due to the hardware cost and complexity, a wearable would not be able to take full advantage of these wireless technologies. Thus, due to the requirement of low energy consumption, no bright use of cellular wireless technology is observed in the perspective of wearable, body-worn WBAN devices. However, in the future, we hope to have such technology for the wearable, body-worn types of WBAN applications.

In addition to discussing these wireless technologies in detail, for simplicity and quick review, the summarized features of these technologies are also given in Table 3 [3].

### 2.4. Application of Wearable WBANS

**Figure 8:**
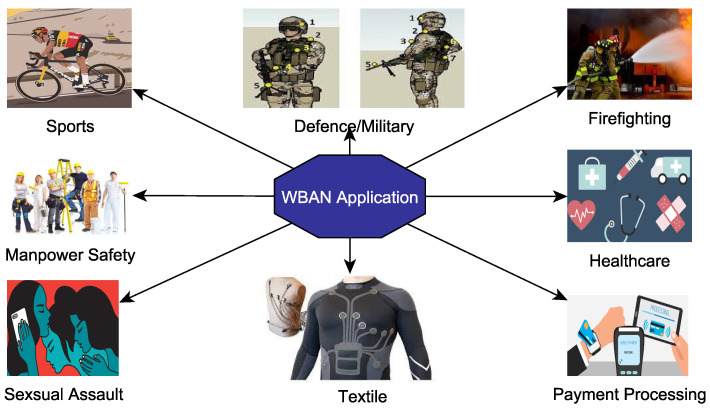
Wearable, body-worn applications.

The applications of WBANS, body-worn or wearable, range from medical, health, fitness, defense, and military to sports and entertainment. A simple view of the different applications of wearable/body-worn devices is given in Figure 8. Research communities believe in the great acquisition of wearables in medical health care, military, and sports, as they have undoubtedly been proven to bring about positive shifts in these and similar domains of life. These devices are extremely helpful in rehabilitation, monitoring and prevention of diseases, reminding patients to take medicine on time, and monitoring daily activities such as eating, sleeping, walking, and running, and most importantly, they provide timely feedback on health parameters to the centralized system [24,89]. Experts are anticipating that in the very near future, we will be able to buy such wireless wearable gadgets in medical stores.

**Figure 9:**
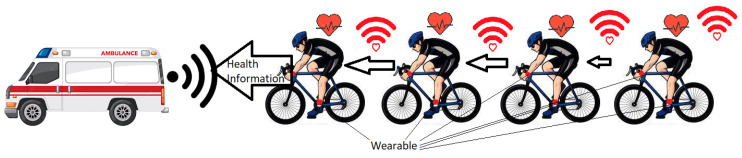
Body-to-Body network [91]: group of wearable wireless body area networks.

Wearable and WBAN are also applied to *Sports and Entertainment* to measure the blood pressure, heartbeat, and temperature of the sportsman along with his state of motion [38,90]. There is a non-medical application of WBAN devices in the entertainment industry that supports real-time audio/video streaming, gaming, virtual reality, etc., available in the form of smart MP3 players, smart microphones and smart cameras [38,90]. *BBN* stands for Body-to-Body network, which is a group of wearable WBANs that communicate with one another. These WBANs are usually connected with a gateway, which plays an important role in sharing of data among these WBANs. It is a theoretical mobile ad hoc network that is emerging as a key technology for sharing data between patients and medical teams [91]. If we consider the sports field, this application can be easily deployed with the help of smart devices such as smartwatches, smart glasses, and smart helmets that can be used to perform estimation and prediction of athlete health. The simple model of BBN is shown in Figure 9. In this figure, every cyclist has a WBAN; all these BANs are connected to the ambulance.

**Figure 10:**
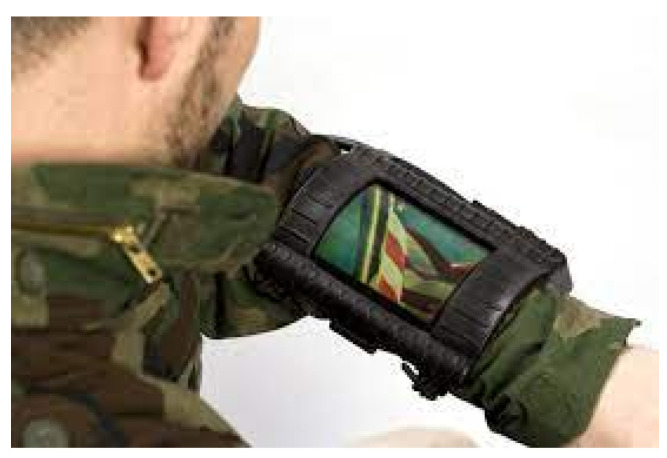
Wrist band with a 4.3 inch flexible OLED display [93].

With respect to disaster incidents, such as fires, earthquakes, cyclones (e.g., Tsunamis), tornado, bomb blasts, etc., it is believed that wearable WBANs is one of the technologies that could help people make themselves prepared for such incidents; equipping them with the tools to react against such incidents in a more effective way [92]. For example, rescue teams could be fitted out with wearables, which could gather and transmit back the exact environmental condition (such as heat, the toxicity of gases, lightning, fire, smoke, etc.) and life-critical information (such as BP, HR, body temperature, etc.). *Military and Defence* applications of WBANs, wearable and body-worn, include the detection of life-threatening factors regarding soldiers and assessing the fatigue thresholds of the soldier [90], whereas WBAN devices are being used for fire, injurious, smoke, and gas detection, which could prevent the death of military men in danger [38]. The most recent development of a wrist band with a 4.3 inch bendable OLED (Organic Light Emitting Diodes) display has transformed fiction into a reality. The wristband has been tested by the US army. It was funded project of the US Department of Defence to develop a thin lightweight and flexible communication device. This device has the capability to show different sources of information such as Unmanned Air Vehicle (UAV) video and images received through a computer that runs different military applications. Moreover, the device is also capable of reducing risks on the battlefield while improving the operational efficiency of troops at the same time. The rest of the technical detail can be viewed from [93]. For motivation, the band is shown in Figure 10.

Usually, people are aware of wearable BAN devices that are used by civilians, but they know less about the same types of products being used for research in the military. There are different military projects, such as “The Army’s Program Executive Office”, “FLIR SYSTEM”, etc., that have developed or are endeavoring to design more sophisticated products. There is a very good system study on the analysis of military-based wearables, which is carried out by Han et al. [94]. Some of the military projects and their products are given in Table 4.

#### Vital Sign Monitoring

WBAN is a network of body-worn or wearable computing devices that can be fixed inside the human body or can be implanted or worn on the body. With respect to vital sign monitoring, WBANs have two different types of uses in the *healthcare sector*; one is medical and the other is non-medical. For medical purposes, they are deployed to measure ECG, EEG, EMG SPO_2_, BP and drug injection [90]. Devices are also implanted in the body to control different diseases such as diabetes. WBAN devices are being used in non-medical areas for motion detection, for example, walking, running, jogging, etc. [38,95]. However, in the case of *manpower safety*, wearable WBAN not only monitors the vital signs such as BP, body temperature, pulse rate, and remaining energy but also carry out motoring of life-critical environment parameters such as carbon dioxide and carbon mono-oxide, ammonia, heat, humidity, etc. [92]. Wearables are classified into different categories based on their application in the real environment such as smartwatches, fitness trackers, smart clothing, smart jewelry, head-mounted devices, smart glasses, wearable cameras, location trackers, body sensors, implants, exoskeleton, and gesture control wearable [5,96]. For simplicity and brevity, wearables are categorized into different types.

The major smart and state-of-the-art vital sign monitoring solutions are Visi Mobile System, Aingeal, VitalSense-VS100, Zensor, QUASAR, EQ02 LifeMonitor, VitaPart-3, PERFORM, Physiodroid, etc. [97]. For more simplicity, details of these devices are given in Table 5, and their detail can be viewed from the respective reference.

In addition to vital sign monitoring devices mentioned in Table 5, there are a lot of other useful wearable and WBAN devices, which are making human lives healthier. The devices in this list may be Google Glass, Withings Scale, Sense Mother, Moto 360, Septimu Earbuds, Smart Diapers, Electronic Fork, etc. [122]. Some of the leading wearable hardware and application developers, manufacturers, and software developers are Fitbit, Medtronic, Nokia, and Philips [123]. Fit-Bit-Flex, Jawbones-Up, Nike+FuelBand, Spire are a few of the top listed wearable solutions among the popular fitness trackers. All these devices have two following common functionalities.

1. They use sensors to determine heart beat rate, walking or running steps and number of calories burned.
2. These devices can be connected to a computer, laptop, or mobile to communicate the results wirelessly.

In addition to these two common functions, Fit-Bit-Flex can also track (i) how many hours a person slept the previous night, (ii) the food intake, (iii) how much exercise one has performed, etc. Nike+FuelBand can track sleep hours in addition to the functions mentioned above. Similarly, Jawbones-Up is a good choice to improve sleep habits and achieve dietary goals as well. It can be interfaced with a number of third-party applications, such as MapMyFitness, Sleepio, MyFitnessPal, RunKeeper, etc. Aspire, however, is one of the most expensive devices that determines a person’s fitness based on the measured patterns of breathing instead of the activities. Garmin is one of the leading manufacturers of body-worn and wearable devices. It has launched different smartwatches, specially focusing on the fitness and performance of different sportsmen, such as divers, sail-men, pilots, and aviators, etc. [124]. Therefore, it can be concluded that wearable WBAN devices are accelerating the growth of wireless communication equipment at a rapid rate.

It is observed that most users are naive to the physiological values given by medical wearables. Some results of medical wearables, such as the values of ECG, are life-critical, and only the expert can comprehend and deduce concrete results. That is why the purpose of this study is not to make people capable of comprehending such complicated results of the medical wearable. However, on the other hand, some readings are simple, and the user can understand, comprehend and take some measures such as BP, HR, temperature, oxygen absorption, hydration and sugar level, and fall distance, etc. Therefore, the normal ranges of these physiological parameters are given in Table 6. In the absence of medical staff, the users can detect abnormalities and take necessary first aid action on the basis of measured and reference values. These values are important and must be known to the common person, which is why they are extracted and reproduced in this article instead of referring the reader to [125]. The readers who belong to the medical domain may refer to [126,127] for an in-depth study of human physiology, but it is beyond the scope of our study.

### 2.5. WBAN Security Issues

Applications of wearable WBANs are discussed in Section 2.4, and the scenarios similar to these applications put wearable WBANs at higher risk of being attacked by adversaries. The broadcast nature of wireless networks plays a major role in exploiting and compromising them. Because these devices carry military and life information, swift security systems must be developed to prevent data leakage, hacking and stealthy ransomware attacks [123].

#### 2.5.1. Major Security Requirements

In addition to the device and user authentication, security is built upon a triad CIA that is a combination of three pillars. These are the major security requirements that any communication is required to fulfill, i.e., confidentiality, integrity, and availability, as discussed below.

1. *Device Authenticity:* This is a very critical issue. Adversaries can implant a malevolent node in a non-line-of-sight (NLOS) location that can inject fake data and even receive legal data while acting as a legal device [128]. Thus, it is very important to validate the authenticity of the BAN devices that they claim to be [129].
2. *Confidentiality:* Confidentiality ensures that the information should not be disclosed to unauthorized entities while moving from wearable WBAN source to destination [130]. However, due to the open and broadcast nature of WBAN transmission, the data can be eavesdropped and disclosed to an unauthorized party. Therefore, it is always advised to encrypt data before transmission [128].
3. *Integrity:* It claims that information must reach the destination in its original form as was sent by the body-worn or wearable WBAN source; it must not be altered during its transfer. Due to the free-flying of wireless BAN frames, they can be intercepted and tampered with by the attacker, which may harm the health system or even the patient’s life [128,130]. That is why it is required to ensure the integrity of data using some message authentication techniques such as hashing or CRC. They ensure the data are not modified during the transition from one BAN device to another.
4. *Availability:* One of the most disturbing attacks is DOS (Denial of Service), which is launched against a body controller unit, cloud server, or medical server. Thus, the attack can even cause a shutdown of medical services, which may impose serious life threats on patients [128,130]. For ensuring life safety and timely treatment of the patient, health network communication systems must be available whenever a WBAN source is required to send data.
5. *User Authentication/Authorization:* In remotely controlled healthcare WBAN systems, only the authentic and authorized medical staff members must be able to access the data of a particular patient. User authentication can be established using well-informed mechanisms, such as username/password, PINs, fingerprints, etc. Similarly, a proper access control mechanism (e.g., Access Control Lists) must be devised to ensure authorized access to data [129].

#### 2.5.2. Importance of Security

We have seen a number of wireless technologies being used in the WBAN industry for life-critical data collection and dissemination. If such information is stolen, it can be used for illegal activities that could result in losses to the owner in the shape of mental disturbance or financial blackmailing. For brevity, a generic set of WBANS attacks, security requirements and measures have been given in Table 7 [131,132,133,134].

Another perspective of the attacks and issues is to view them from a WBAN network stack point of view. We have observed from [131,133] that jamming and tampering are major issues at the physical layer. On the link layer, collision, unfairness, denial of sleep, traffic analysis, spoofing, eavesdropping and exhaustion are some of the issues that affect the security of WBAN devices. Similarly, the network layer also has to face different attacks such as selective forwarding, blackhole, rushing, wormhole, sinkhole, sybil, homing, neglect, misdirection, etc. Flooding and desynchronization are the most havoc attacks that are usually projected on the transport layer of the WBANs. Some of the solutions that are recommended to take control measures on the physical layer are frequency hopping, spread spectrum, tamper-proofing, and link hiding. Similarly, the link layer can be protected against mentioned security issues by using error correction codes, rate limitation and adaptation, or making use of small frames. For the protection of network layer probing, packet filtering, authorization monitoring and encryption can be used. Similarly, energy-efficient secure routing protocols are required for the long life of WBAN networks [135]. Client puzzles and authentication techniques can be used to protect from flooding and desynchronization issues on the transport layer. For a detailed review of these attacks, a reader is advised to read [136]. Unfortunately, due to its wireless nature, WBANs are highly susceptible to different network threats and attacks. For simplicity, these attacks have been categorized into active and passive. Passive attacks are designed just to listen to network traffic, whereas active attacks can make harmful changes to the WBAN data in transition or storage. Some of the common attacks are eavesdropping, data corruption and modification, replay, masquerade and impersonation [74]. WBAN wireless protocols have been reported to face different attacks, which are summarized in Table 8.

#### 2.5.3. Technical Approaches

Encryption is a basic tool to provide confidentiality and integrity. It is achieved through the encryption key, which is a shared secret between the source and the destination. It is widely believed that security lies in the secret key’s size and in its strength, which means how much a secret key is random or uncertain in its nature. Presently, two or more nodes can agree upon a shared secret using state-of-the-art protocols such as DH (Diffie–Hellan), ECDH and RSA [154,155]. These techniques are just computationally secure methods with a sufficient amount of resources, and the adversary can breach the security implemented by DH, ECDH and RSA. Furthermore, PKI technology does not suit resource-constrained networks such as WBANs, body-worn or wearable. It has been observed that in some scenarios, wireless wearable body area network technology is very sensitive, and the information in these cases is highly classified and critical. That is why the protection of information and keeping it consistent for different stakeholders and users is very important. Different state-of-the-art security solutions are unleashed in this article to save the sensitive information of wearable WBAN technology. For more clarity, the security support of different wireless wearable BAN technologies is discussed individually and separately.

##### TinySec Security Solution

Wireless technology is inherently insured due to its broadcast nature. TinySec is a link layer security architecture for wireless sensor networks and is the first fully implemented security protocol for these networks [156]. It ensures authentication, integrity, and confidentiality. However, it does not deal with resource consumption, tampering and node theft attacks. For access control and integrity, it makes use of MAC (Message Authentication Codes) and encryption for privacy, and the encryption keys are pre-configured. It makes use of single a network-wide, group, and pair-wise secret key for network, group and peer–peer communication. Instead of using software-based encryption as in the case of TinySec, hardware-based encryption can also be coded with the help of ChipCon-2420 ZigBee compliant Radio Frequency (RF) transceiver. The ChipCon-2420 can execute security operations of Zigbee 802.15.4 with AES using a secret key of length 128-bit [157].

##### ECC

Elliptic Curve Cryptography (ECC) is one of the suitable choices for public key encryption for wireless body area networks. This suits WBAN because its small key size has been proven to be highly secure. In addition to this, it also generates a small signature. Moreover, tremendous amount of computation power is required to breach ECC. Such computation power will not exist until quantum computing becomes a reality. However, it cannot be ignored that ECC demands a significant amount of energy compared with other contemporary approaches, which provides an alternative solution for high system security [158]. An energy efficient solution based on ECC for WBAN was presented by Tianchan et al. [159]. It is worth mentioning that there are different types of encryption schemes such as symmetric key encryption, public key encryption (e.g., identity-based encryption (IBE), and attribute-based encryption (ABE)). From the list of public key-based authentication types, ABE and IBE are well-known techniques for encryption purposes. For the security of WBANs, attribute-based encryption was presented by Tian et al. [160], whereas ID-based encryption was proposed by Chu et al. [161] for wireless sensor networks. IBE reduces the computational costs of encryption and decryption.

##### Biometric-Based Security Solution

This is quite an innovative approach for providing security solutions in low-power wireless devices. Encryption is one of the most accepted ways of protecting information that needs encryption and a decryption key. A symmetric key can be established among different BAN or wearable devices. In the biometric-based security method, the human body endeavors to achieve a secret key by using EEG/ECG values measured by different body sensors, which are attached or embedded in different parts of that body. Since all EEG/ECG devices observe similar values, they can agree on a shared symmetric key between them [162,163,164]. This key, later on, can be used in encryption and decryption processes.

##### Encryption

**Figure 11:**
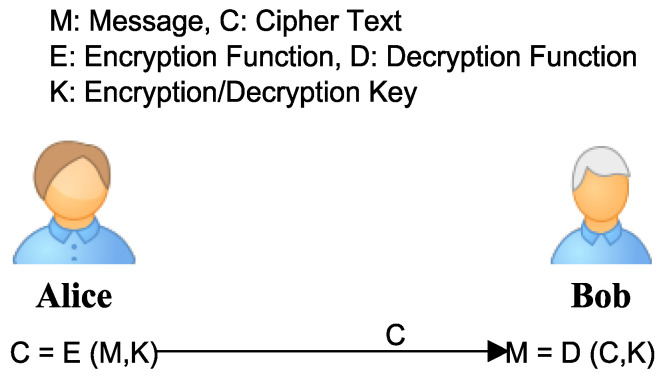
Symmetric key encryption.

WBAN and wearable technology can be protected with the help of different encryption mechanisms, which may provide a high level of security using symmetric, asymmetric, and identity-based encryption. *Symmetric Encryption* is a type of encryption in which symmetric keys are used for encryption and decryption. For simplicity, Figure 11 shows a message that is encrypted and decrypted by two parties, Alice and Bob, using an identical shared secret.

**Figure 12:**
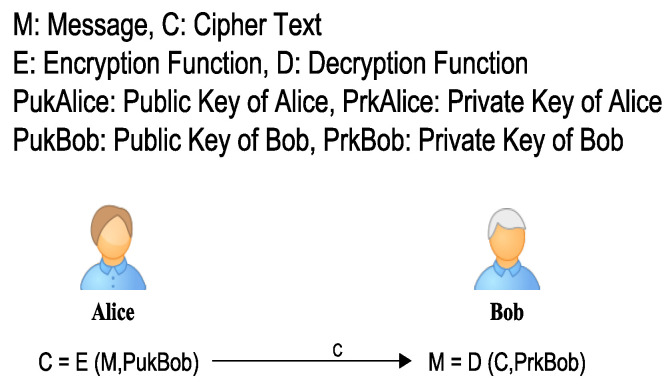
Asymmetric key encryption.

*Asymmetric Encryption* is a type of encryption in which two different keys are used for the encryption/decryption process. One key is used for encryption, while the other is used for decryption. This mechanism is also called Public Key Cryptography, where each party holds a pair of public/private keys. The sender encrypts a message with the public key of the receiver. The receiver upon receiving the encrypted message used its private key to decrypt it. Public keys are shared, while private keys are kept secret and are not disclosed to anybody. Figure 12 shows that each encryption and decryption key is different, i.e., Alice encrypts a message, with the public key of Bob and Bob decrypts the encrypted message with his private key.

IBE is the singleton of ABE in the sense that IBE uses a single attribute to link a secret/private key with a public parameter, e.g., email that works as an ID. In this case, the owner of the secret/private key can decode the message, whereas ABE makes use of multiple attributes. e.g., name, rollno, class, city, etc., and links these attributes with a secret/private key. The owner of the private key who matches these attributes can only decrypt the message encrypted by a sender.

#### 2.5.4. Wireless Security Fixes

##### ANT/ANT+/ANT+S

ANT is a network protocol that is designed for IoTs. Like the other protocols, such as 802.11, it also operates in the 2.4 GHz frequency band. ANT+ is based on ANT and was developed by Dynastream Innovations. By default, ANT+ is an unencrypted protocol. However, it provides security with the help of RF Frequency/Channel ID and network key. Communication on a unique channel-ID or frequency prevents legal devices from receiving data from unintended devices. However, it does not stop illegal devices from malicious interception of legitimate communication. A 64-Bit network key is used to encrypt ANT+ packets, but this size does not provide adequate security. Security threats can be overcome with ANT+s optional encryption, that is, AES-128 in CTR mode. Discussing ANT+ data acquisition and processing are goals of this article but can be seen from [165].

##### RuBee IEEE 1902.1

RuBee is the commercial name of IEEE 1902.1, which is known as Long-Wavelength ID (LWID) [166]. RuBee provides a secure physical communication layer, flexible asset flow layer, and asset visibility. It provides secure asset flow and security while not depending upon encryption only. It has four layers that are used to implement the security of visible assets and data packets. These layers are (i) Asset Flow Layer, (ii) Physical Asset Layer, (iii) Packet Data Layer and (iv) Physical Communication Layer. Layer-i and layer-ii deal with the secure flow of visible assets, whereas layer-iii and layer-iv handle the secure flow of information. Moreover, RuBee is capable of providing a controlled physical layer security with almost no interceptions. Similarly, it is not affected by water, steel, or other harsh environmental factors. Rubee tags support the packet security layer using advanced encryption standards such as AES and Public/Private Key Cryptography [167].

##### Sensium

Sensium is a wireless radio protocol specially designed and optimized for vital sign monitoring. It is a robust, reliable and secure wireless patient monitoring system. It is implemented in the form of a miniature and disposable patch [168]. Sensium makes use of the public key cryptography for information security [169].

##### Z-Wave

An early version of Z-Wave employed S0 security, which was vulnerable to eavesdropping, message manipulation, and injection. However, a later version of security S2 was quite a lot stronger due to leveraging the strength of the Elliptic Curve Diffie–Hellman (ECDH) curve called Curve25519 and a public key length of 256 bits. S2 is also based on AES-128-CCM for authenticated encryption and AES-128-CBC for secret key derivation [170].

##### Zarlink

Zarlink makes use of Reed-Solomon Codes with CRC (Cyclic Redundancy Check) for establishing a reliable communication link [76].

##### Insteon

Insteon provides CRC and FEC (Forward Error Correction). It provides a higher level of security with a unique addressing mechanism (unique ID code), AES encryption, and secure channel allocation. Actually, it has multiple encryption methods, such as rolling-code, managed-key and public-key [169,171].

##### LR-WPANs IEEE 802.15.4 Security Services

LR-WPANs stands for Low-Rate Wireless Personal Area Networks. The link layer security protocol of 802.15.4 supports four basic features, which are access control, data integrity, data privacy, and data protection from replay attack [131,172].

##### WBAN IEEE 802.15.6 Security

IEEE 802.15.6 is accompanied by three different security levels, which are (i) Unsecured Communication Level, (ii) Authentication Level, (iii) Authentication and Encryption Level. Level-1 provides no security, Level-2 supports only authentication, while Level-3 comes up with authentication and encryption [173,174]. Moreover, the master key is always pre-shared; temporary pairwise and group keys are generated as per the communication requirement. A detailed security analysis of IEEE 802.15.6 can be reviewed from [175].

##### ZigBee Security Services

Zigbee is lower power wireless technology that establishes an ultra-lower communication setup in an existing spectrum of a wireless world. Zigbee inherits the security services of 802.15.4 and adds extra features to an existing set. The extra services provided by Zigbee at the network layer are in the form of authentication and key exchange. Zigbee network nodes make use of network and link keys for communicating messages secretly. Receiving nodes always know what keys are used for securing the messages [176]. The current standards of Zigbee provide a trust center that is an application that allows the zigbee nodes and router to be configured and authenticated for joining the network [177]. Zigbee security is good, but ZiCoskun et al. [178] showed the security comparison of Zigbee, Bluetooth, and NFC; they concluded that Zigbee and Bluetooth security levels are lower than NFC.

##### BLE Security

BLE stands for Bluetooth Low Energy, and it uses periodic transmission for small data packets. The older versions of this protocol such as BLE 4.0, and BLE 4.1 used a simple procedure connection in which BLE devices exchange a temporary key that is used to generate a short-term secret key. This short-term key is used for the authorization of the connection. These connections are not secure but can be made secure with the help of a protected pairing process. In BLE 4.2, secure connections were introduced, which are not compatible with the legacy version. Elliptic-curve Diffie–Hellman (ECDH) is used to generate a more complex secret key, which introduces a robust process of key authentication [179]. It prevents passive eavesdropping. BLE devices can be further secured with the help of a secure pairing process [180].

##### Bluetooth Security Protocols

Classic Bluetooth protocol stack consists of different protocols such as Baseband, Logical Link Control and Adaptation (L2CAP) and Link Manager Protocol (LMP), Service Discovery Protocol (SDP). The establishment of a link between different Bluetooth devices and the exchange of information using packets is the responsibility of a baseband protocol. LMP protocol induces security features such as encryption, authentication, and key exchange in Bluetooth. As far as the L2CAP is concerned, it provides support for multiplexing at a higher level. This protocol also supports reassembling of packets that, in turn, support quality of service. Similarly, SDP is used to discover services that are hosted on all Bluetooth devices. Jini and UpnP are a few service discovery protocols that can be used in combination with the SDP protocol. Bluetooth operates in three modes: (i) silent: device only monitors traffic and is undiscoverable, (ii) private: device is undiscoverable and accepts connections from only known MAC-Addresses, (iii) public: device is discoverable and accepts connection from any device [181]. A careful selection of an operating mode also elevates security. In addition to these operating modes, Bluetooth supports four security modes whose functionalities are given below [146,181].

1. Non-secure: No security.
2. Service-level enforced security mode A non-secure ACL (Asynchronous Connection Link) can be established using this mode. Security functions run only after the L2CAP connection-oriented or connection-less channel request is placed.
3. Link-level enforced security mode Security primitives are started before making any request for a channel is initiated and the ACL link is established.
4. Service-level enforced security mode (SSP) This is similar to service-level enforced security mode; the only difference is that only the Bluetooth devices that use secure simple pairing (SSP) can be set in this mode.

Security comparison of Zigbee, Bluetooth, and NFC can be seen from [178], which indicated that NFC provides better security than Zigbee and Bluetooth.

##### 802.11 Security

Since the discussion on different versions of 802.11 is beyond the scope of this study, we used 802.11 security protocols irrespective of the 802.11/a/b/g/n. The most popular security protocols that have been used to protect 802.11 networks are Wired Equivalent Privacy (WEP), WiFi Protected Access (WPA), WiFi Protected Access version 2 (WPA-2), and WiFi Protected Access version 3 (WPA-3). WPA-2 is also known as 802.11i. WEP was the original security protocol developed in the late 1990s for achieving confidentiality. Due to security flaws, in 2003, WiFi Alliance declared it superseded by WPA. In 2004, with the ratification of WPA-2, WEP was set to phase out by the same alliance. WEP used RC-4 for authentication and encryption. WPA also uses RC-4 stream cipher for encryption, but it is enhanced with the help of Temporal Key Integrity Protocol (TKIP). WPA-Extensible Authentication Protocol (WPA-EAP) is an enterprise mode that uses 802.1x for authentication. Whereas in personal mode (WPA-PSK), it makes use of pre-shared keys for security purposes. WPA-2 is more robust and reliable; it uses Advanced Encryption Standard (AES) instead of RC-4. It uses CCMP protocols for authentication [182]. CCMP stands for Counter Mode with Cipher Block Chaining Message Authentication Code Protocol. WPA-3 is considered to be the most recent and secure 802.11 security solution. In 2018, WiFi Alliance started looking for the certification of WPA-3, and by July, 2020, they inquired about devices seeking certification of WPA-3. WPA-3 provides support of security for personal and enterprise networks. WPA3-Personal provides natural password selection, ease of use and forward secrecy. WPA3-Enterprise provides a very high level of security using advanced cryptography algorithms. Authentication is achieved by using EAP; AES-CCMP 128-bit is used in encryption. For key derivation and verification, 256-bit HMAC-SHA is used. Similarly, for robust frame protection, 128-bit Broadcast/Multicast Integrity Protocol Cipher-based Message Authentication Code (BIP-CMAC-128) is used. WPA3-Enterprise with 192-bit is optional and provides more advanced level of security with the combination of a selection of security primitives [183].

## 3. WBAN Economical Issues

### 3.1. WBAN/Wearable Compound Annual Growth Rate

The global market of wireless wearable BAN devices is usually assessed with the help of CAGR, which stands for Compound Annual Growth Rate. It is defined as the annual growth rate of investment between two years, assuming the growth is increasing exponentially and one year profit is reinvested in the next year. CAGR is one of the most accurate ways of calculating returns on investment that can increase or decrease over a period of time. Equation (1) is used to calculate CAGR [184]. The purpose of explaining CAGR in this article is that most computer, material and life scientists and engineering professionals do not know the concepts of commerce.

(1) $$CAGR=\left({\left(\frac{EV}{BV}\right)}^{1/n}-1\right)\times 100$$

where *EV* and *BV* are ending and beginning values, and *n* is the number of years. We believe this brief explanation of CAGR can help the reader to understand it better from the perspective of global investment in wireless wearable body area networks. It is forecasted by “Transparency Market Research” that the global body area network market will touch USD 62,120.9 Million in 2025. This information was broadcasted by Cision PR Newswire who the mentioned market is expected to grow at the CAGR rate of 24.7% from 2017 to 2025 [185]. According to recently reported information by James Hayward [186], the market of wearable BANs devices was $80 Billion in 2020, which was thrice the market of 2014 in terms of annual revenue. It is predicted that the market of wearable BANs could reach USD 138 billion by 2025 [187]. According to a very recently published technical survey on wearable technology, 20% annual growth rate of this technology is expected, and its market is predicted to touch EUR 150 Billion by 2028 [5]. It can be analyzed from the shipment of wireless wearable BAN devices, which was EUR 113.2 million in 2017 and was estimated to reach EUR 223.3 million every year by 2021. One of the driving factors was COVID-19 during this period [5]. As for the global enterprise market of wireless wearables is concerned, alone it was over EUR 18 billion in 2017, which is estimated to grow at a CAGR of 11.8%, taking 2017 as the beginning and 2026 as the ending year [5,188]. Similarly, a business research organization named “360 Research Reports” published its analysis result in June 2021, which forecasts that the global market of wireless wearable body area networks, which was at USD 11950 million in the year 2021 will increase with the compound annual growth rate of 6.6% from 2021 to 2027 [189]. In addition to this, an organization named “Market Research Future” also forecasts that the market of body area network devices can rise up to USD 21 billion at a CAGR of 13% by 2025 [190,191].

**Figure 13:**
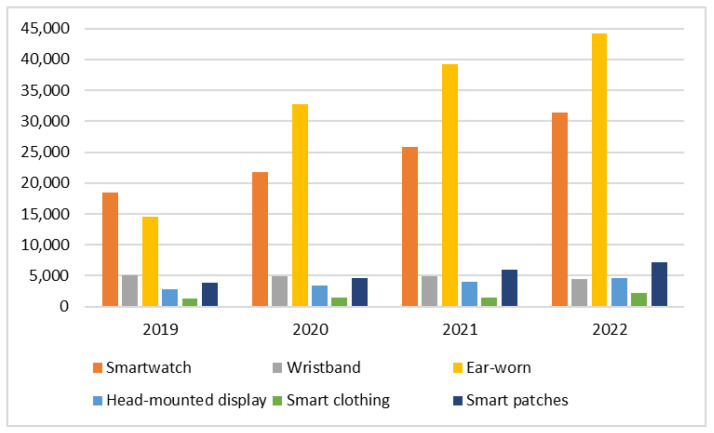
Consumer spendings on wearables [192].

Return on investment in the wearable BAN industry can also be assessed from a report recently released by a leading provider of wearable BAN devices named Gartner [192]. The report shows a continuous increasing trend in the use of wearable devices, as shown in Figure 13. The report discloses that the spending on ear-worns rose from USD 14,583 million to USD 32,724 million from 2019 to 2022, and it is predicted that it will rise from USD 39,220 million to USD 44,160 from 2021 to 2022. It is observed that consumer spending on Smartwatch increased from USD 18,501 million to USD 21,758 million during the period of 2019 to 2020, and it has a chance to rise from USD 25,827 million to USD 31,337 million from 2021 to 2022. According to this report, smartwatches and bands collectively captured 51% share of the market in 2019. In 2021, ear-worns were the leading wearable with a market share of 48%, and smartwatches and wristbands are at the second number with a collective market share of 37%. Similar trends are predicted for the year 2022 in this report.

**Figure 14:**
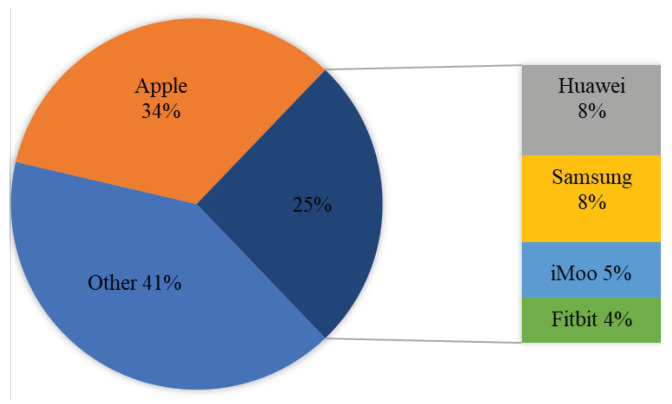
Year 2020 major players of smartwatches [193].

Fortunately, we have data available about the major wearable global market players that show that Apple is the top shareholder in the market of smartwatches [193]. According to the reported data, CAGR of the wearable BAN market could be seen as 19.48% from 2019 to 2026. Figure 14 shows the share of a few other players as well.

**Figure 15:**
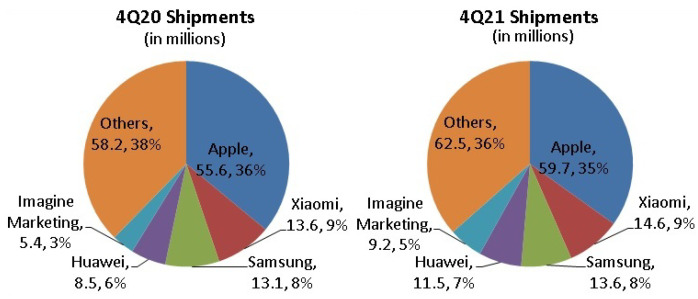
Year 2020 and 2021 major players of wearable shipment [194].

Similarly, IDC Corporate USA reported that Apple retained the first position with about 34.9% of the wearable shipment market share from 2020 to 2021 (4^rth^), whereas Xiaomi, Samsung, Huawei, Imagine Marketing, and others had an 8.5%, 7.9%, 5.6%, 5.4% and 36.6% wearable market share in the fourth quarter of 2021 [194]. Wearables that were included in the survey is earwear, watches, wristbands, and others. The information from 2020 and 2021 is given in Figure 15.

**Figure 16:**
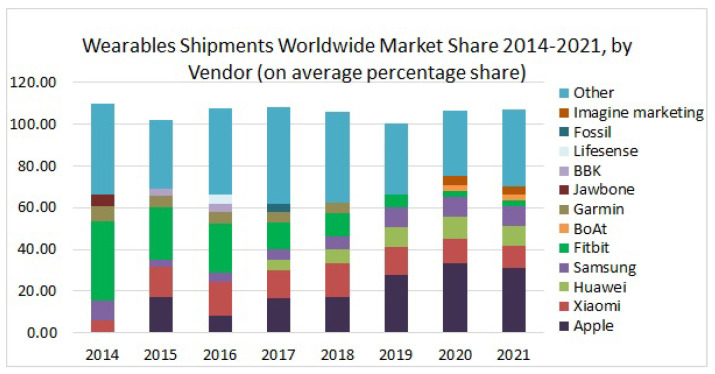
Year 2020, 2021 Major Players of Wearable Shipment [195].

Similar to a business survey on wearable major players presented in [194], Federica Laricchia [195] published a similar report but with greater coverage of wearable vendors and the time span, i.e., from 2014 to the third quarter of 2021. Year-wise average percentage shipment share of wearable devices is shown in Figure 16, but complete quarter-wise information can be obtained from [195].

### 3.2. WEARABLE/WBAN Regulatory for Body

It has been observed that people know less about the regulatory bodies and their guiding principles regarding medical devices. That is why we decided to include this section as a part of this study. The production, shipment, and uses of wearable BAN devices are increasing exponentially; due to this, manufacturers are required to comply with different factors or parameters of health, environment, and data protection laws, standards, and regulations. In addition to this, they are supposed to follow the different regulatory requirements of wireless connectivity, cybersecurity, product safety, chemical ingredients of the product, electromagnetic compatibility, device interoperability, one-time-use of plastic, e-waste, disposal, and many more [196]. Due to the exponential growth of wearable BAN devices across the world, their range and complexity have also increased. This growth rate triggered the evolution of more stringent and robust policies, standards, rules and regulations for wearable BAN devices. They must be obeyed by the manufacturers. However, it has been observed that the regulation of such devices significantly varies all around the world, which is very strict to none [197]. These regulations help manufacturers sell their products in multiple countries of the world that follow predefined sets of rules and regulations for wearable BAN and medical devices.

**Figure 17:**
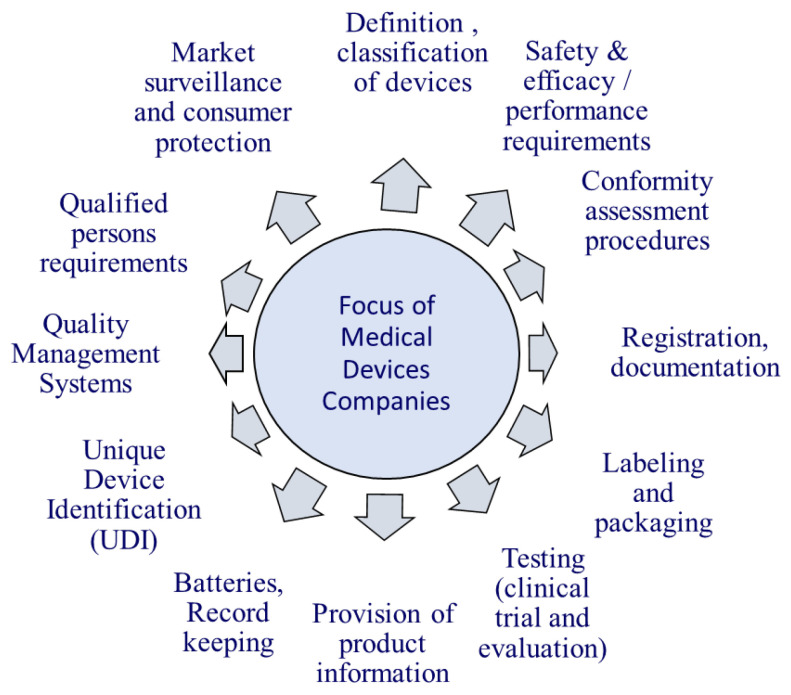
Focus of medical device companies [196].

The purpose of developing standards, policies, rules and regulations is to ensure the safety of consumers because they are not capable of deciding which medical device to buy, which to use, how to use it, and how to assess the potential risks behind the use of particular device or medicine [196]. It means the BANs medical devices cannot obtain success if they do not comply with the regulations and standards set by regulatory and standardizing authorities. That is why device manufacturing companies focus on different requirements and parameters of health-related devices in light of guidelines that are provided by regulatory bodies and authorities. The generic names are given to such requirements, as shown in Figure 17. However, respective stakeholders such as manufacturers or importers can obtain information about standard requirements, guidelines, rules/regulations, and procedures, from the respective regulatory authority of that region for manufacturing, launching, promoting/marketing, and disposing-off of such wearable BAN devices. Regulatory authorities or standardizing organizations provide all sorts of technical documents that are required to be submitted to activities. Furthermore, the devices may be medical or non-medical.

It is not possible to discuss the rules and regulations of different regulatory bodies that are active in different countries of the world for regulating the manufacturing, use, and disposal of wireless wearable BAN devices. Therefore, we have decided to only provide authentic and useful links to different regulatory organizations and their guidelines, policies, standards, rules, and regulations. For instance, Canadian rules and regulations for medical devices are devised and consolidated by Medical Device Regulation (MDR). They are published by the Canadian health minister on the official law website of the Govt. of Canada [198]. This is a very recent document that was updated on 20 October 2021. It is a very useful document for the manufacturers of wearable medical BAN devices because it contains the latest guidelines concerning the regulation of such devices. Regulatory bodies are working on regulations in which a manufacturer should submit a single report that must meet the requirements of all regulatory bodies. There are different authorities who are responsible for the regulation and standardization of health-related devices (medical or non-medical), which may be wireless wearable body area network devices or they may be conventional medical equipment. Authority-wise, major multinational regulatory bodies are given in Table 9. In addition to this, country or region-wise, they are given in Table 10. There are some organizations that cover multiple countries or states, but they are placed in Table 9 considering the limitation of their application areas. This is just for the ease of a reader; otherwise, it does not matter too much whether they are placed in Table 9 or Table 10.

### 3.3. WEARABLE/WBAN Feasible Projects

Human life is always in the process of improvement and betterment. From this study, we have come to know that wireless wearable BAN devices are playing a vital role in this regard. However, the researchers are still working hard to design and develop more sophisticated devices for the well-being of human life. One such effort is the recent development of OvulaRing, Vitalpatch, FreeStyle Libre, and Zio XT^®^ [74]. OvulaRing is for doctors and ladies; it can find the most suitable time of fertility. VitalPatch can measure HR (Hear Rate), RR (Respiratory Rate), and temperature. It can also detect falls and perform ECG. Similarly, FreeStyle Libre is implanted under the skin; it can monitor sugar levels. Similarly, Zio XT^®^ can detect irregular activities of the heart.

One of the leading projects in the direction of a future wearable is H2020 A-WEAR ITN/EJD. It is a four-year project (2019–2022) whose target is to educate, train and supervise 15 researchers to face the future challenges of wearable technology. This project is funded by European Union’s Horizon 2020 (H2020) Marie Sklodowska-Curie Innovative Training Networks H2020-MSCA-ITN-2018 call https://projects.tuni.fi/a-wear/, accessed on 14 April 2022 [5].

Hospital Without Walls is another attempt made by Australia’s Commonwealth Scientific and Industrial Research Organization (CSIRO) whose target is to provide continuous vital sign monitoring information of the person to medical experts through telephone lines or using the internet. This healthcare system makes use of miniature low-power wearable radio technology to transmit health information to the home server and then to suitable medical experts using WAN technology [227].

The pace of wearable technology can be imagined from the data that are being uploaded on PatientsLikeMe72 and the evolution of self-governed healthcare community projects such as Nightscout and the Open Artificial Pancreas73, which gives a clear indication of future research and development perspectives of a wireless wearable healthcare system [228].

Selective information has been extracted from the article written by Luis et al. [229] and Rahat et al. [74] and given in Table 11. The military projects have not been added to this table as their information is already provided in Table 4.

## 4. Open Issues

### 4.1. Research Challenges

Wireless wearable body area networks encounter some interesting challenges that might be easily resolved in resource-enriched networks but difficult to resolve in low-power devices such as wearable and BANs. These issues greatly affect the efficiency, deployment and social acceptance of wireless wearable BAN devices. They are briefly discussed below to attract the attention of scholars, scientists and researchers so that they may address them while designing the security and privacy solution for wireless wearable BANs.

#### 4.1.1. Security vs. Efficiency

For data security of wearable and WBANs, efficiency is one of the most stringent requirements. However, due to limited resources for computation, storage and data rate, it is very hard to achieve high efficiency. That is why it is recommended to make use of lightweight cryptographic functions as much as possible for data security. These functions must be low-cost in terms of processing, memory, transmission and power consumption because complex security primitives may lead to high computational load, which may also lead to early drainage of battery [5,38].

#### 4.1.2. Security vs. Safety

It is very difficult to prioritize between security and safety, especially in emergency conditions when the patient is unconscious and cannot respond. Very strict security in terms of accessing patient data may prevent the medical staff from acquiring it well on time. Thus, it can create an adverse situation when giving medical treatment to the patient. However, at the same time, loose and flexible access control may open back doors for malicious users to illegally play with the patient’s medical information with evil intentions in mind. Therefore, it is difficult to provide good security with loose data access rights.

#### 4.1.3. Security vs. Usability

The users of wireless wearable BAN devices may not be experts, which is why these devices must be easy to operate, configure and integrate with other interfaces such as mobiles, laptops, cloud servers, etc. In order to avoid human errors, it is required that there must be a minimum possible human interaction in data security procedures. For example, the technique of automatic device pairing can be used to establish initial secure communication between wireless wearable BAN devices. Similarly, it is well-advised to provide security services at different phases of data collection, processing, storage, and retrieval.

#### 4.1.4. Device Interoperability

Device interoperability is one of the key factors for the social, technical, and industrial acceptance of the wireless wearable WBAN devices [5]. There might be different vendors and manufacturers of these and similar devices. However, the devices must be compatible and interoperable with one another. For example, it is appealing when someone connects a Samsung Galaxy health monitoring Watch-3 with an Apple iPhone [230]. The views of wearable and BAN users might be adverse due to the lack of device interoperability.

#### 4.1.5. Device Authentication

In addition to the data or message authentication, device authentication in the case of wearable and BAN devices is also one of the critical issues that have attracted significant attention from security researchers. Devices claiming their legitimacy by rendering that they are honest and belong to the same wearable/BAN network need validation. Different research studies are observed in the literature regarding BAN device authentication meaning whether a claimant really hosted on the same body or not. However, this area is still in its infancy and requires more research work.

#### 4.1.6. Secret Key Acquisition

We do not advocate the employment of public key cryptography algorithms for low-resource wearable BAN as they are resource-hungry and conflict with the limited resources of devices. We believe in unleashing new methods of secret key acquisition compared with traditional DH key exchange protocols. Different wireless information-theoretic security approaches are proposed that exploit the natural process of the physical and MAC layers for establishing the shared secret. The shared secret can be used for authentication and encryption.

#### 4.1.7. Strict Regulations

Wireless wearable BANs have to pass through the strict regulation of the FCC and other health regulatory and standardization bodies.

### 4.2. Research Directions

The field of wireless wearable body area networks calls for different open research contributions to improve its usage. Key research challenges are given below.

1. *Global RF Design:* The Radio Frequency of the Wireless Wearable BAN must be such that the devices can operate in any part of the world because it is observed that some wireless channels are allowed to operate in the USA but not in other parts of the world or some channels are used in one part of the world but not in other. Thus, RF design must be able to cope with heterogeneous wearable BAN devices [38].
2. *Performance:* Other high-power ISM wireless devices operating in the surrounding may degrade the performance of low-power wearable and WBAN devices making them less attractive and appealing for high-quality medical and healthcare applications. For instance, Wireless Medical Telemetry Service (WMTS) frequency bands are extensively being used, but unfortunately, their use is limited to healthcare applications in the USA. Moreover, UWB is a good candidate technology for wireless wearable devices, but at the same time, it is badly affected by the interference of the co-existence of high data rate applications such as wireless multi-media.
3. *2360–2400 MHz Band:* Federal Communication Commission (FCC) has opened the 2360–2400 MHz band for medical WBAN. It is believed that the band will be used to devise an artificial nervous system to reinstate sensation and mobility and regain the functionality of paralyzed limbs and organs. However, the devices based on this medical WBAN frequency band are required to test and validate against the FCC health-related rules and regulations [231]. Therefore, designing devices that exactly meet the FCC regulations is also one of the great challenges.
4. *Channel Model Design:* Another research challenge is the designing of the RF channel model because it plays an important part in the designing of PHY (Physical Layer) technologies. PHY is an abbreviation of the Physical Layer of TCP/IP or OSI Model. PHY is implemented to connect the MAC layer with the physical medium; in this, it is a wireless Radio Frequency (RF). PHY consists of RF, Transceiver, DSP (Digital Signal Processor), communication protocol, and channel codes. PHY is integrated with MAC in SOC (System on a Chip) technology. Examples of wireless PHY are 3G, 4G, LTE, WiMAX, and UWB.
5. *Radio Antenna Design:* Designing an antenna for wireless wearable BAN devices is also very critical due to restrictions on size, shape and choice of material [232,233]. Since the devices are wearable, patched on the body or implanted inside the body, the size of these antennae must be miniaturized. Designers do not have too many choices for the shape of antennae; it is the organ that puts restrictions on the shape. The material that can be used to design antennae should be harmless for the human body such as titanium or platinum. However, antennae made of such material result in low performance when compared with the antennae made up of copper.
6. *Physical Layer Protocols:* Physical layer protocols of wearable and BAN devices must assure low power consumption without compromising reliability, connectivity, throughput and turnaround time [5,38]. Fast wake-up from sleep mode and speedy turnaround time can significantly save the power of wearable/WBAN devices. Presently, energy efficiency is being achieved through duty cycling, but researchers are endeavoring to find new ways of energy saving such as low-power listening harvesting [234].
7. *MAC Design:* It is always required to have a wearable BAN MAC (Medium Access Control) layer that should be resilient to interference experienced due to the co-existence of multiple WBANs in crowded areas such as hospitals. MAC layer protocols should be adaptable to frequent topology changes. Moreover, MAC protocol of the WBAN should be able to switch to a silent channel in the case of severe interference due to the co-existence of similar networks operating in the surrounding using the same frequency band [38].

### 4.3. Emerging Techniques in WBAN

#### 4.3.1. Human Body Communication (HBC)

After IoTs, a new concept is emerging in BANs called Internet of Bodies (IOBs) that makes use of conductive features of our tissues to make human body communication, but completely benefiting from IoBs is still a topic of hot discussion related to risks and challenges, such as HBC channel modeling [22].

#### 4.3.2. Energy Harvesting

It is one of the emerging concepts in WBANs, which, in addition to working on power optimization, aims to extend the battery life by harvesting light/solar, motion, thermal or RF energy [235,236].

#### 4.3.3. Quantum Computing

It is believed that quantum computing will solve many problems in large-scale WBAN calculations such as magnetic resonance imaging (MRI), computed tomography (CT) and nuclear medicine (e.g., positron emission tomography (PET)) [235].

#### 4.3.4. Graph Neural Networks (GNN)

Recently, GNN has had wonderful secure applications in social networks, recommendation and knowledge systems and life sciences. In computer science, a graph is a data structure in which the two components are named vertex and edge. GNN is a neural network that operates on a graph structure to produce desired results. In wearable WBANs, they are being used for human action recognition [237,238].

### 4.4. Artificial Intelligence: Machine and Deep Learning

Machine learning is one of the key emerging technology for processing health data for diagnosis purposes and is playing an important role in enhancing the performance of WBANs [239,240]. Communication networks are also benefiting from technology [241].

#### 4.4.1. Blockchain

Blockchain is a distributed database shared among the different nodes of the network. Blockchain is an innovative technology in the sense that it ensures the security of records and assurance of trust without using a third party. WBAN data can be stored and protected using blockchain [242,243]. Blockchain integration with WBANS, its trends and future opportunities can be seen from [243,244].

#### 4.4.2. Digital Twins

It is an outstanding technology that simulates what-if scenarios for products, and processes that need to be changed and tested before deploying into the real/physical world [245]. Digital racing is one of the best examples that can be given to understand this concept. Digital twin technology is being applied in the healthcare industry for developing new methods of diagnosis and prognosis of remaining life, tear and wear and injury locations, etc. [246].

## 5. Conclusions and Future Work

The following are a few points that conclude our present study concisely.

1. Wireless wearable BAN devices are one of the marvelous inventions in human history that improves the apex quality of human life.
2. There is great investment potential in the global industry of wireless body area networks that have also been observed from the different CAGR values reported in many global market analysis reports.
3. On the other hand, a lot of work is still required to make those devices whose manufacturing ingredients involve radio or electromagnetic waves and frequencies safe for the living body cells and tissues.
4. Wireless body area networks consist of very low-resource devices, which is why it is demanded that communication and security protocols must be light in computation, storage, and transmission because these factors are directly linked with the lifetime of BAN nodes as they are battery powered.

While considering the resource limitations of wearable wireless BAN devices, it is highly desired to look for security measures that must be lightweight with respect to time (processing), space (memory), and messaging (bandwidth). That is why, in the future, we have planned to use some probabilistic space-efficient data structures, such as hyperloglog, count-min sketch, bloom filters, cuckoo hashing, to achieve device authentication, data confidentiality, and integrity. We believe our future research work will prove to be a groundbreaking step in the acquisition of secret keys and carrying device authentication not only in wearable WBANs but also in IOTs.

## Figures and Tables

**Table 1:** Diversity of WBAN sensors used for life sign monitoring.

Sensor	Implantation	Observation
Guardian Real-time	inside the body	Sugar
MiniMed 530G with Enlite	wearable, injectable	Sugar
FreeStyle	inside the body	Sugar
Eyeglasses biosensor	body-worn	Sugar
Eyeglasses biosensor	body-surface	Sugar
Guardian Real-time	inside the body	Sugar
IFever	body-worn	Temperature
ISense	body-worn	Temperature
Ran’s Night	body-worn	Temperature
FeverFrida thermometer	body-surfaced	Temperature
STEMP sensor	body-surfaced	Temperature
Aranet medical thermometer	body-worn	Temperature
Propeller	body-worn	Asthma
AirSonea	body-worn	Asthma
Wing	body-worn	Asthma
BH1790GLC	body-worn	HR
TCRT 1000	body-worn	HR
Smart QHeart Rate Sensor	body-worn	HR
Polar H10	body-worn	HR
Pulse sensor	body-worn	HR
MPU-9250 Invensense	body-worn	Motion
MMA 8451Q, accelerometer	body-worn	Motion
LSM 303 DLHC	body-worn	Motion
BNO055	body-worn	Motion
MPU-9250 Invensense	body-worn	BP
Biosignals plux	body-worn	BP
Proven BPM-337BT	body-worn	BP
Omron M7 Intelli IT	body-worn	BP

**Table 2:** LoRaWAN security features [85].

Scheme	Standard LoRaWAN	Improved DO-SEO [85]
Mutual Authentication	✓	✓
Secure Key Exchange	✓	✓
Perfect Forward Secrecy	✕	✓
End-to-End Security	✕	✓
Thwart Replay Attack	✕	✓

**Table 3:** Wireless protocols adopted by wearables industry.

Protocol	Distance Range	Data Rate	Power Consumption	Frequency Band	Suitability
ANT (from Garmins)	100 m outer door	1 Mbps	15 mA Tx 17 mA Rx	2.40–2.483 GHz	used in sports, fitness and health devices
RuBee (IEEE 1902.1)	30 m	9.6 k	emits energy in nanoWatts, battery lasts for many years	operates below 450 kHz	considered to be a strong contestant for WBAN application
Insteon	30 m	9.6 k	Battery life is 10+ years	operates below 450 kHz	considers being a good protocol for WBAN applications
Sensium	home area	13 k	5 days	131.65 KHz, 902–924 MH	extensively used in health care and diagnostic application, worthy of medical implanting cases, gastrointestinal [74].
Bluetooth Classic (IEEE 802.15.1)	100 m	1–3 Mbps	2.5–100 mW	2400–2483.5 MHz range within the ISM 2.4 GHz frequency band	used in different area of tele-medicine and e-health systems, but unfit for WBANs [70].
BLE (Bluetooth 4.2)	50–150 m	1 Mbps	10 mW	2.4 GHz	But, not suitable for frequent and steady reporting of health parameters [70]. At the
IEEE 802.11	150–200 m	54 Mbps	1 W	2.4 GHz (WiFi), 5 GHz	encouraging wireless technology for WBANs [70]
Zigbee 3.0, 802.15.4	10–100 m	250 kbps	35 mW	2.4 GHz mostly; though some devices use 784 MHz (China), 868 MHz (Europe), 915 MHz (USA, Australia)	unsuitable for critical WBAN application which are required to carry urgent data without delays [70].
LoRa (LoRa Alliance)	urban 2–5, suburban 15, rural 45	0.3–50 kbps	(customizable), battery life more than 10 Years	169 MHz, 433 MHz, 868 MHz (Europe) and 915 MHz (North America)	used as LPWAN [88]
NFC (ISO/IEC 18000-3)	10 cm	106, 100–420 kbps	15 mA or less	13.56 MHz	smart suits, jackets, jewelry, wrist worns, e-skin, [49]
Z-wave	30 cm	106, 212 or 424 kbps	it uses 23 mA in transmission mode, battery lasts for decades even	900 MHz (ISM), (Z-Wave Alliance ZAD12837/ITU-T G.9959)	considered to be a good protocol for WBAN applications [74].
SigFox	2–5 (urban), 15 (suburban), 45 (rural)	10–100 kbps	battery life more that 10 years	433/868/780/915 MHz	used as LPWAN, no mobility and location support [88]
NBIoT	2.5–5 km	more than 250 kbps	battery life more than 10 years	Cellular (Licensed Band)	used as LPWAN, needs GPS for location support, mobility [88]
LTECatM1	2.5–5 km	1 Mbps	battery life more than 10 years	Cellular (Licensed Band)	used as LPWAN, needs GPS for location support, supports mobility [88]
WBAN (802.15.6)	suitable for short-range human body communication	MICS (75–455, 75–187, 101–607), $ISM_{1}$ (101–607) Mbps, $IMS_{2}$ (121–971)	0.1 μW	MICS (402–405, 420–450, 863–870), ISM_1_ (902–928, 950–958), ISM_2_ (2360–2400, 2400–2483.5)	Most of the medical, non-medical, and on-body WBAN devices have adopted this standard for communication, support for location and mobility

**Table 4:** Military wearables.

Product	Project	Product Type	Features
Life- BEAM	LifeBEAM	Helmet	Monitors beating heartrate using optical sensor
Combat- Connect	The Army’s Program Executive Office	Wearable electronics system	disseminate information to/from WBAN using smart hub fixed with military vest/jacket
Black Hornet	3 FLIR SYSTEM	Automatic Pocket-sized helicopter	camera that can be fixed with armor/vest; works as elevated binocular
Ground Warfare Acoustical Combat System	Gwacs Defense	A wearable tactical system	Identifies and locates hostile fire; detects and tracks Small UAVs
Exo- Atlet	ExoAtlet	Lower body powered exoskeletons	Mobility assistant, reduce troop’s movement cost
SPaRK	Spring Active	Energy-scavenging exoskeletons	Performs energy harvesting to recharge devices

**Table 5:** Vital sign monitoring systems.

Monitoring Device/System	Vital Signs	Provider	Ref.
Visi Mobile System	It is (Continuous Beat-by-Beat Blood Pressure) CNiBP, Non-Invasive Blood Pressure (NiBP), ECGs, Respirations, HR, PR, SPO2, Motions and Postures. Visi Mobile System continuously leveraging on hospital’s existing WiFi infrastructure Sotera Wireless	Sotera Wireless, Inc.	[98]
Mobile Cardiac Outpatient Telemetry (MCOT) Patch System	MCOT collects medical information using Bluetooth from sensors, transmits ECG wirelessly to BioTel Heart all the time	Bio-Tel Heart	[99,100]
MCT 3 Lead (MCT 3L) Cardiac Telemetry	LifeWatch MCT 3 is very smart in nature, giving a distinct picture of the heart in addition to the rhythms of a heartbeat.	Bio-Tel Heart	[99]
ePatch Extended Holter Monitoring	Records and stores heartbeats	Bio-Tel heart	[99]
wEvent	Wireless Cardiac Event Monitor, wEvent supports arrhythmia detection with high precision	BIO-Tel Heart	[99]
Wireless Connector for BPA-060	Wireless Blood Pressure Monitor	Ambio Health	[101]
Wireless Connector for Presto	Blood Glucose Monitor	Ambio Health	[101]
WiCap	Capnography device, monitors expired (EtCO2), inspired (FiCO2) breath, respiration rate (RR), SpO2, and pulse rate (PR)	Athena GTX	[102]
Tac Pac	Measures NIBP, HR, SpO2, and ECG	Athena GTX	[102]
Athena Device Management Suite (ADMS)	ADMS connects different wireless vital-sign monitoring devices and is also adaptable to mobiles and smartphones, alarming, awareness, etc.	Athena GTX	[102]
Wireless Vital Signs Monitor	Wirelessly monitors Sp02, HR, NIBP, and ECG within a range of 200 yards, ADMS compatible	Athena GTX	[102]
Caretaker	measure CNiBP, Heart Rate, and other vital signs, android compatible	Caretaker Medical	[103]
SmartSense	Temperature, blood pressure, pulse, SpO2, respiration, pain level, SMD, etc., via any mobile PC, draw plots of vital signs	Cadi Scientific Pte Ltd.	[104]
HealthPatch/Vital Patch, Vista Solutions	Health monitoring system, which can measure HR, RR, temperature, posture, activity, and fall detection	Vital Connect	[105]
Fitbit Premium+Health	It is a personalized health monitoring system that helps a person manage stress, have a sound sleep, and stay active. It provides a daily score of these signs. Users can download their data at any time	Fitbit LLC.	[106]
Garmin Connect	Garmin Connect can track, analyze and share your health data on mobile or web	Garmin Ltd.	[107]
Samsung Health	Galaxy smartwatch-based health solution that scores sleep and blood oxygen during sleep. It can also detect snoring and monitor running performance	Samsung Ltd.	[108]
Apple Health Kit	It assesses the steadiness of walking, falling probability, and goodness of sleep that help helps to assess changes in health. The health data can be shared with concerned people	Apple	[109]
Wireless Implantable Cardioverter and Defibrillators	Cardioverter and defibrillators give weak and strong electrical shocks to the heart to maintain rhythm and beating. FD approved such devices a long time ago [110,111]. Each manufacturer has introduced its remote monitoring system, pacemakers and ICDs, such as Home Monitoring^TM^, CareLink Network^TM^, Latitude Patient Management system^TM^, and Merlin.net^TM^ (St Jude Medical, Sylmar, USA). [112]	Biotronik, Medtronic, Boston Scientific, Merlin.net	[113,114,115,116]
EEG, ECG, EMG, EOG, EGG monitoring devices such as Bittium Faros^TM^—Cardiac Monitoring	These devices can measure ECG, EMG, EEG, etc., TSE Systems, Lepu Medical Technology, Bittium Corporation, BIOPAC Systems, Inc. are major providers. Stellar Implants, NEO ECG, and Mobita are also good monitoring systems	Fabric-embedded devices available at [117]	[118,119,120,121]

**Table 6:** Normal ranges of commonly known physiological parameters [125].

Parameters	Normal Range
Body Temperature	37–37 Degrees Celsius
Hear Beat	Rate 60–100 beats per minute
Breathing Rate	12–20 breaths per minute
Oxygen Saturation	94–99%
Blood Sugar	4–6 mmol/dL (72–108 mmol/dL)
Blood Pressure	120/80
Hydration level	90–100%
Fall Detection	0–20 cm

**Table 7:** Security threats and possible solutions in WBAN [131,133].

Security Issues	Secure System Requirements	Potential Security Measures
Illegitimate access to information	Dynamic secure key agreement and trust establishment	Random ephemeral key generation and distribution, use of public key cryptography
Information Disclosure	Confidentiality and privacy	Link/network layer encryption and access control
Data Modification	Integrity and authenticity	Keyed secure hash function and Digital signature
Denial of Service	Availability	Intrusion detection systems and redundancy
Compromised/Hijacked Node	Cryptosystem must be resilient to node-compromise	Anomaly detection, comprising nodes isolation
Routing vulnerabilities	Protection of routing information	Use of secure routing algorithms
Intrusions/Unauthorized Access to Network System	Intrusion detection and prevention, secure data aggregation, group communication and management	Intrusion detection and prevention systems

**Table 8:** Attacks on WBAN wireless technologies.

Protocol	Security Risks	Proposed Control Measures
ANT	by default ANT+ is not encrypted, AES128 support in special cases, provided in single channel communication only, lack of authentication, MITM [137].	network key along with network number provides different options for access control and security level [137,138]
Rubee	no eavesdropping, high packet security due to date and time stamp [139].	eavesdropping range is similar to that of tag range; such a close eavesdropper can be detected easily, it meets a high standard of security also being used in many US Govt. owned departments [139] of USA
Sensium	Sensium is vulnerable to all those attacks, launched on body sensor networks, such as insider attacks, DOS, wormhole, replay, jamming, etc. [140]	per hop encryption can reduce the chances of attacks, also reduces overload over the network as well [140]
Zigbee	insufficient integrity protection due to sending unencrypted key; location privacy, an initialization vector (or Nonce), and key management are susceptible to high-security risks; improper support of group keying [141]. Due to default weak password, misconfiguration of the Zigbee IP network, and the gateway or lack of security an attacker can bypass Zigbee authentication check [142]; replay attack, device identification attack, and packet interception are a few of the other ones [143]	time stamping, AES, and Message Integrity Codes (MIC) can be used to mitigate the risks [143]. In addition to this, since the security keys are stored in memory, adversaries can directly steal them. Therefore, it is advised to use a microcontroller for secure authentication and to eliminate the threat of physical corruption of security keys [144].
Bluetooth	intrusion in pico-net, MITM (Man-in-the-Middle)/Impersonation, DOS attacks, Bluejacking [142,145]. A few other reported attacks are MAC-spoofing, PIN cracking, BlueSnarfing, BlueBugging, BluePrinting, Blue over, off-line PIN recovery attack, brute-force, reflection, backdoor, Cabir worm, Skulls worm, and Lasco worm [146].	use long PIN Codes, do not pair in public areas, and turn on non-discoverable mode after use, at the corporate level proper policies and guidelines be devised for Bluetooth pairing [145]
WiFi	cipher attack is used to attain the encryption key through processing information exchanged between sender and receiver. WEP, WPA-PSK Dictionary, WPA/TKIP, and LEAP are a few of the cipher attacks. WiFi also faces, jamming, flooding, evil-twin threats [142,147]	separating the guest network from actual, encrypting traffic with WPA2 or WPA3, updating firmware timely, creating secure SSID, restricting WiFi to business hours only, all these measures can mitigate the attacks on WiFi [148].
LORA	attacker can obtain access to physical LORA device, jamming-communication channel can block with the high power radio signal, replay, beacon synchronization, traffic analysis, MITM are a few threats to LORA [84]. DOS, Bit-flipping, ACK spoofing. LoRa vulnerability survey can be viewed from [149,150].	dynamic or ephemeral key generation, *DevNonce* of large size can prevent join attacks [151], Block-chain is also good for powered limited devices [88]. Use Over-The-Air-Activation (OTAA) [150]
NFC	eavesdropping, cloning of ticket, spoofing, a man in the middle attack, corruption of data, insertion of malicious data, relay, ghost and leech, DOS, phishing, a threat to ID, PIN, credentials of credit card, etc.	shared secret keys, certificate-based authentication WEP based security can be used to reduce the probability of these attacks [73]
Z-Wave	Z-Wave device pairing process with the controller can run many times, which attracts “key reset attack” [142,152]	vulnerability has not been fixed, more than one million devices are in the market with this vulnerability [152]
WBAN	Key-Compromise Impersonation (KCI), Impersonation, WBAN security protocols do not forward secrecy as well [153]	using certified keys KCI can be avoided, but it is not recommended in standards due low resources of WBAN devices [153]

**Table 9:** National and international regulatory bodies.

Regulatory Body	Year and Location	Members	Comments
WHO [199]	1948, Geneva (Switzerland)	Globe is a member of the Word Health Organization (WHO)	Looks into global health concerns. https://www.who.int/, Accessed on: 14 April 2022
Global Harmonization Task Force (GHTF), its website is ceased, visit [200]	1992, USA	USA, European Union (EU), Japan, Australia and Canada, AHWP, International Organization for Standardization (ISO), and the International Electrotechnical Commission (IEC)	Volunteer, the original group of people from MDR formed it for the regulation of medical. GHTF was replaced by International Medical Device Regulators Forum (IMDRF) in 2012 with the aim build a strong foundation of GHTF. https://iris.paho.org/bitstream/handle/10665.2/51549/PAHOHSS19003_eng.pdf, Accessed on: 14 April 2022, https://www.adb.org/sites/default/files/publication/184392/better-regulation-medicine.pdf, Accessed on: 14 April 2022, https://pdf.usaid.gov/pdf_docs/PA00TPBN.pdf, Accessed on: 14 April 2022
IMDRF [200]	1911, NA	Australia, Brazil, Canada, China, Europe, Japan, Russia, Singapore, South Korea, USA	Successor of GHTF
ISO [201]	1947, Geneva Switzerland	163 countries are members International Organization for Standardization (ISO)	largest organization in the world for formulating standards https://www.iso.org/home.html, Accessed on: 14 April 2022
Emergency Care Research Institute (ECRI) [202]	1968, Pennsylvania USA	USA, European Free Trade Association (EFTA), But USA and Canada mutually developed Universal Medical Device Nomenclature System (UMDNS)	ECRI maintained UMDNS, the development of Asian Medical Device Nomenclature System (AMDNS) is also based UMDNS https://www.ecri.org/, Accessed on: 14 April 2022
APEC [203]	1989, Singapore	Australia, Brunei Darussalam, Canada, Indonesia, Japan, Korea, Malaysia, New Zealand, the Philippines, Singapore, Thailand, USA, China, Hong Kong, Chinese Taipei (Taiwan), Mexico, Papua New Guinea, Chile, Peru, Russia and Vietnam	Asia-Pacific Economic Cooperation (APEC) provides forum for free trade among member countries https://www.apec.org/, Accessed on: 14 April 2022
Asian Harmonisation Working Party (AHWP) [204]	1996–97, Hong Kong (Asia)	Abu Dhabi (UEA), Brunei Darussalam, Cambodia, Chile, China, Chinese Taipei (Taiwan), Hong Kong SAR, India, Indonesia, Jordan, Saudi Arabia, Korea, Laos, Malaysia, Myanmar, Pakistan, Philippines, Singapore, South Africa, Thailand, and Vietnam	It ensures the regulation of medical devices in Asia, now working on Safety Alert Dissemination System. http://www.ahwp.info/, Accessed on: 14 April 2022
Association of Southeast Asian Nations (ASEAN) [205]	1967, Jakarta (South Asia)	Bangkok, Indonesia, Malaysia, Philippines, Singapore, Thailand, Brunei Darussalam, Vietnam, Laos, Myanmar and Cambodia. Within ASEAN The Medical Device Product Working Group (MDPWG) is responsible for the regulations of medical devices.	The ASEAN Medical Device Directive (AMDD) formulate basic requirements for the safety, performance and efficiency of medical devices that include a device classification and post-marketing alert system that should be circulated throughout the ASEAN. https://asean.org/, Accessed on: 14 April 2022
NICE [206]	1999, UK.	European Union. UK is a member of The National Institute for Health and Care Excellence (NICE)	regulates the use health technologies and clinical practices in the UK. Also provides guidance on health care services and promotion. https://www.nice.org.uk/, Accessed on: 14 April 2022
ETSI [207]	1988, Sophia Antipolis, France	European Union	ETSI regulates technical standards of ICT (Information and Communications Technologies) enabled devices, systems and services https://www.etsi.org/, Accessed on: 14 April 2022
FCC [208]	1934, USA	US States comply with Federal Communications Commission (FCC)	FCC regulates radio, TV, satellite and cable communication in USA. FCC and other regulatory bodies look into the issues such as how much radio frequency/ energy absorption is harmful for humans https://www.fcc.gov/, Accessed on: 14 April 2022
HIPPA [209]	Act of 1936, USA	US States follows Health Insurance Portability and Accountability Act (HIPPA)	In the USA, HIPPA is responsible for ensuring that the privacy, integrity and access controls are meeting defined standards, rules and regulation. https://www.hipaa.com/, Accessed on: 14 April 2022

**Table 10:** Country-wise regulatory bodies.

Country	Regulatory Body	Year	Purpose
USA	The Food and Drug Administration [210]	1906, Maryland (USA)	oversees radio and non-radio based medical devices, medicines and drugs, cosmetics, and biologics in the USA
Russia	Roszdravnadzor (Federal Service on Surveillance in Healthcare and Social Development	2004, Moscow (Russia)	it handles registration, clinical safety and efficiency of medical devices. It also oversees post-market surveillance of these devices
Russia	Gosstandart (Federal Agency for Technical Regulation and Meteorology)	2004, Moscow (Russia)	ensures that imported devices meet all Russian standards. Its parent body is Ministry of Industry and Trade
Russia	Rospotrebnadoz (Federal Service for Supervision in the Area of Consumer Rights and Welfare Protection)	1922, Moscow	ensures that BAN devices meet hygienic and epidemiological regulations
China	State Food and Drug Administration (SFDA), China Food and Drug Administration (CFDA) in 2013 China: Also a member of AHWP and APEC	CFDA (2013), NMPA (2018), Beijing (China)	accountable for the regulation of locally manufactured and imported BAN medical devices. CFDA was renamed as National Medical Products Administration in 2018
Hong Kong	Device Administrative Control System (MDACS), Hong Kong is also also a member of AHWP and APEC	2004, Hong Kong	The Medical Device Division (MDD) is accountable for implementing MDACS and developing a long-term statutory regulatory framework for local and imported medical devices. The old name of MDD was Medical Device Control Office (MDCO) formed in 2004.
India	Central Drug Standards Control Organization (CDSCO) India: Also a member of AHWP, Based on The Drugs and Cosmetics Act 1940 and Rules 1945, India	NA	works under Ministry of Health and Family Welfare. Carries regulation of medical devices, imported implantable, diagnostic kits and sterile devices require registration under the current system, registration will follow the GHTF, Central Licensing Approval Authority (CLAA)
Iran	Ministry of Health and Medical Education—Food and Drug Administration, National Medical Device Directorate [211]	NA, NA	device classification follows GHTF, registration is similar to FDA or EU.
Israel	Medical Device Division of the Ministry of Health (AMAR) [212]	NA, NA	devices regulation strictly follows US FDA and EU CE.
Jordan	The Jordan Food and Drug Administration (JFDA) [213], Jordan is also a member of AHWP	2003, Amman (Jordan)	enforces laws and regulation for medical device, if the device is approved from USA FDA and EU CE it does need clearance in Jordan
Saudi Arabia	The Saudi Food and Drug Authority (SFDA) [214], Saudi Arabia is also a member of AHWP	2003	only those devices are permitted that have approval from any one of the GHTF member
UAE	UAE Ministry of Health Drug Registration and Control Department [215], United Arab Emirates (UAE) is also a member of AHWP	NA, NA	UAE adopts guidelines of GHTF, EU, Australian TGA and USA FDA in addition to UAE Pharmacy Law No4 for 1983
Africa	National Medicines Regulatory Authorities (NMRA) [216,217], African Organization for Standardization (ARSO) [218], The African Network for Drugs and Diagnostic Innovation (ANDI), African Union (AU) and United Nations Economic Commission for Africa (UNECA) [219]	ARSO (1970, Kenya), UNECA (1958)	Only 7% of African countries have NMRA, 63% have minute regulations, and 31% are without regulations. ANDI was developed in phases by WHO in collaboration with TDR, AFRO and EMRO, African Development Bank, etc. [220]. More on regulatory authorities in African and East African countries can be seen from [221,222].
Caribbean	Pan American Health Organization (PAHO) [223], also operates in Central and South America	1902, Washington DC	PAHO has assigned a task to ECRI to develop and coordinate programs for medical device safety and regulation
South America	Mercado Común del Sur (MERCOSÚR) [224], Argentina, Brazil, Paraguay, Uruguay, Venezuela Chile, Bolivia, Colombia, Ecuador and Peru	1991, Montevideo (Uruguay)	regulation for medical devices, single approval scheme similar to EU, Products with FDA/EU marks having Free Sale Certificate (FSC) or a Certificate to Foreign Government (CFG) affirm they can be sold
Mexico	Federal Commission for the Protection against Sanitary Risk (COFEPRIS) [225]	2001, Mexico	regulation of medical devices and IVDs. Mexico is also a member of APEC and HAFTA
Cuba	left for the State Control of Medicines, Equipment and Medical Devices (CEMED) [226], Cuba is also a member of GHTF	NA, La Habana	regulation of medical devices based on GHTF. CCEEM is in La Habana (Cuba)

**Table 11:** Wireless wearable body area networks projects [74,229].

Project	Application	Communication
Body Inertial Sensing	Meant for hospitals, where it provides information to measure three degrees of freedom	Body Sensor Networks (BSN) ZigBee/Bluetooth
Code Blue	Targets medical care to provide pulse rate, BP, and motion information	Intra-BAN (Wired), Inter-BAN (Zigbee, Mesh), Beyond-BAN (N/A)
LOBIN	Measures HR, BP, Temperature	Wireless Sensor Networks (IEEE 802.15.4)
MEDISN	Used in hospitals for emergency detection	WSN for monitoring patient physiological data and multi-hop wireless backbone for carrying these data
Unobstructive BAN	Applied in hospital and disaster events to generate alarms based on identified movements and postures	Intra-BAN is Low-power and short-range WBAN, Inter-BAN is Bluetooth, and or Beyond BAN is GSM
WPWS	Measures temperature, humidity, and velocity of air flow	RFID like system
BASUMA	Targeted application area is health monitoring for measuring ECG, Respirate/Breath Rate SpO_2_	Intra-BAN (UWB), Inter-BAN (N/A), Beyond-BAN (N/A)
MobileHealth	Monitoring of patients ECG in ambulance	Intra-BAN (Manual), Inter-BAN (Zigbee, Bluetooth), Beyond-BAN (GPRS, UMTS)
AID-N	Reply BP, ECG, Pulse Rate in emergency systems	Intra-BAN (Wired), Inter-BAN (Zigbee, Bluetooth), Beyond-BAN (WiFi, Internet, Cellular Networks)
MAHS	Targets health application to provide information about breathing and pulse rate, body motion, temperature, pressure and SpO_2_.	Intra-BAN (Bluetooth), Inter-BAN (Zigbee), Beyond-BAN (Internet)
LifeMinder	Targets self-care applications. Used to report pulse rate, ECG, SpO_2_, temperature, body movement, emotional state intensity	Intra-BAN (Bluetooth), Inter-BAN (Bluetooth), Beyond-BAN (Internet)
SMART	Monitor patients ECG, SpO_2_ while sitting in waiting room	Intra-BAN (Wired), Inter-BAN (Bluetooth), Beyond-BAN (N/A)
CareNet	Targets remote healthcare applications to report BP, temperature and body inertial (posture and movement) information	Intra-BAN (N/A), Inter-BAN (Zigbee), Beyond-BAN (Internet, Multi-hop 802.11)
ASNET	Targets remote healthcare applications for providing patient BP, temperature information	Intra-BAN (Wired, WiFi), Inter-BAN (Wired, WiFi), Beyond-BAN (Internet, GSM,)
WHMS	Used in health for monitoring ECG/EKG	Intra-BAN (Wired), Inter-BAN (Wired, WiFi), Beyond-BAN (N/A)
Human++	Provides entertaino-medical living standard with ECG, EMG, EEG facility	Intra-BAN (UWB), Inter- BAN (N/A), Beyond-BAN (N/A)
WiMoCA	Provides gesture detection in sports	Intra-BAN, Inter-BAN (Bluetooth), Beyond-BAN (WiFi, Cellular Networks)
Ayushman	Used in health monitoring and provides information about EKG, BP, SpO_2_ and body gesture	Intra-BAN (Zigbee), Inter-BAN (WiFi), Beyond-BAN (Internet)
Lifeguard	Applied in space and terrestrial applications to monitor ECG, respiration and pulse rate, SpO_2_ and body motion	Intra-BAN (Wired), Inter-BAN (Bluetooth, Internet), Beyond-BAN (Bluetooth/Internet)
IBBT IM3	Used in Telemedicine and Telecare application and measures patient ECG, respiration and heart rate	Intra-BAN (N/A), Inter-BAN (N/A), Beyond-BAN (Internet)
MITHrl	Used in healthcare applications and measures patient ECG and EKG	Intra-BAN (Wired), Inter-BAN (WiFi), Beyond-BAN (N/A)
UbiMon	Used in healthcare application and measures patient ECG and EKG, SpO_2_	Intra-BAN (Zigbee), Inter-BAN (WiFi/GPRS), Beyond-BAN (WiFi/GPRS)
H2020 MCSA ITN/EJD A-WEAR	Used in healthcare application and measures patient ECG and EKG, SpO_2_	Intra-BAN (Zigbee), Inter-BAN (WiFi/GPRS), Beyond-BAN (WiFi/GPRS)

## Data Availability

Not applicable.
